# Therapeutic properties and pharmacological activities of asiaticoside and madecassoside: A review

**DOI:** 10.1111/jcmm.17635

**Published:** 2023-02-08

**Authors:** Shinjini Bandopadhyay, Sujata Mandal, Mimosa Ghorai, Niraj Kumar Jha, Manoj Kumar, Radha, Arabinda Ghosh, Jarosław Proćków, José M. Pérez de la Lastra, Abhijit Dey

**Affiliations:** ^1^ Amity Institute of Biotechnology Amity University Kolkata India; ^2^ Department of Life Sciences Presidency University Kolkata India; ^3^ Department of Biotechnology, School of Engineering & Technology Sharda University Greater Noida India; ^4^ Department of Biotechnology Engineering and Food Technology Chandigarh University Mohali India; ^5^ Department of Biotechnology, School of Applied & Life Sciences (SALS) Uttaranchal University Dehradun India; ^6^ Chemical and Biochemical Processing Division ICAR – Central Institute for Research on Cotton Technology Mumbai India; ^7^ School of Biological and Environmental Sciences Shoolini University of Biotechnology and Management Sciences Solan India; ^8^ Department of Botany Gauhati University Guwahati India; ^9^ Department of Plant Biology, Institute of Environmental Biology Wrocław University of Environmental and Life Sciences Wrocław Poland; ^10^ Instituto de Productos Naturales y Agrobiología (IPNA) Consejo Superior de Investigaciones científicas (CSIS) Santa Cruz de Tenerife Spain

**Keywords:** asiaticoside, cardioprotective, *Centella asiatica*, madecassoside, neuroprotective, skin

## Abstract

*Centella asiatica* is an ethnomedicinal herbaceous species that grows abundantly in tropical and sub‐tropical regions of China, India, South‐Eastern Asia and Africa. It is a popular nutraceutical that is employed in various forms of clinical and cosmetic treatments. *C. asiatica* extracts are reported widely in Ayurvedic and Chinese traditional medicine to boost memory, prevent cognitive deficits and improve brain functions. The major bioactive constituents of *C. asiatica* are the pentacyclic triterpenoid glycosides, asiaticoside and madecassoside, and their corresponding aglycones, asiatic acid and madecassic acid. Asiaticoside and madecassoside have been identified as the marker compounds of *C. asiatica* in the Chinese Pharmacopoeia and these triterpene compounds offer a wide range of pharmacological properties, including neuroprotective, cardioprotective, hepatoprotective, wound healing, anti‐inflammatory, anti‐oxidant, anti‐allergic, anti‐depressant, anxiolytic, antifibrotic, antibacterial, anti‐arthritic, anti‐tumour and immunomodulatory activities. Asiaticoside and madecassoside are also used extensively in treating skin abnormalities, burn injuries, ischaemia, ulcers, asthma, lupus, psoriasis and scleroderma. Besides medicinal applications, these phytocompounds are considered cosmetically beneficial for their role in anti‐ageing, skin hydration, collagen synthesis, UV protection and curing scars. Existing reports and experimental studies on these compounds between 2005 and 2022 have been selectively reviewed in this article to provide a comprehensive overview of the numerous therapeutic advantages of asiaticoside and madecassoside and their potential roles in the medical future.

## 1 INTRODUCTION

**FIGURE 1:**
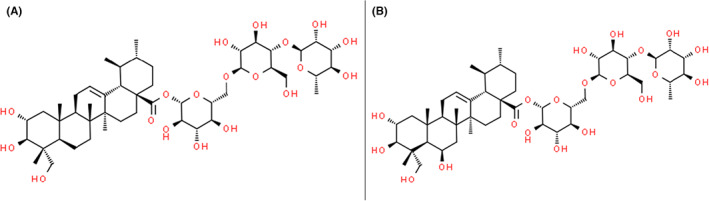
Chemical structures of asiaticoside (A) and madecassoside (B)

*Centella asiatica* (L.) Urban (also known by its common names; Indian pennywort, Gotu Kola) is a medicinal plant belonging to the Apiaceae family. This perennial, herbaceous creeper grows in swampy areas and is native to the Asian tropical regions of the Indian subcontinent, Pakistan, South East Asia, Malaysia, Indonesia, some temperate regions in China, Japan, Korea and Taiwan as well as the equatorial belt of South Africa, Madagascar and South‐Central America.^1^, ^2^ *C. asiatica* is rich in pentacyclic triterpene glycosides (also called saponins or centelloids) and the medicinal efficacy of the plant is mainly attributed to these primary active constituents, asiaticoside and madecassoside (Figure 1, retrieved from www.ChemSpider.com), as well as their respective aglycones (sapogenins), asiatic acid and madecassic acid. These triterpene saponins are common secondary plant metabolites that are synthesized via the isoprenoid pathway to produce a hydrophobic triterpenoid structure (aglycone) containing a hydrophilic sugar chain (glycone) which is responsible for the biological activity of the saponins.^3^ Other compounds derived from the herb include phenolic acids, triterpene steroids, volatile oils, flavonoids, tannins, phytosterols, vitamins, essential oils, amino acids and sugars. The saponins and their aglycones are the most abundant pentacyclic triterpenoids in *C. asiatica* with asiaticoside and madecassoside accounting for roughly 8% of the herb dry mass.^4^, ^5^ Madecassoside occupies the highest concentration among the triterpene saponins in most *C. asiatica* extracts.^5^ However, the quantity of triterpene constituents of *C. asiatica* varies depending on the diverse geographical origin, genetic, environmental and growth conditions.^1^ Table 1 describes different preparations of *C. asiatica* extracts whose compositions include asiaticoside and madecassoside.

**TABLE 1:** Different preparations of *C. asiatica* extracts containing asiaticoside and madecassoside.

Extraction solvent	Plant part(s)	Preparation method	Main composition	Highest triterpene saponin component	Region of plant sample collection	References
Ethanol	Arial parts	Response surface methodology, Dynamic maceration	Madecassoside, asiaticoside, madecassic acid and asiatic acid	Madecassoside	Thailand	148
	Fresh and dried leaves	Microwave‐assisted extraction, Vacuum microwave‐assisted extraction	Asiatic acid, asiaticoside, madecassic acid, madecassoside and phenolics	Asiatic acid	Thailand	149
	Fresh leaves	Microwave‐assisted extraction	Phenolics, flavonoids and triterpenoids	Asiaticoside	India	150
Methanol	Fresh leaves	Soxhlet extraction	Asiaticoside, madecassic acid, madecassoside, asiatic acid	Asiaticoside	Madagascar	151
Methanol/water	Dried plant	Ultrasonic extraction or sonication, Soxhlet extraction, Microwave‐assisted extraction	Asiatic acid, asiaticoside and madecassoside	Madecassoside	China	152
	Dried, leaves	Ultrasonic extraction or sonication	Asiatic acid, asiaticoside, madecassoside, madecassic acid	Asiaticoside	Madagascar	153
Ethanol/water	Fresh and dried leaves	Maceration	Madecassoside, asiaticoside, madecassic acid, asiatic acid, phenolics	Madecassoside	Thailand	154
Methanol, ethanol, deionized water	Dried leaves, nodes, petioles and roots	Subcritical water extraction	Asiatic acid, asiaticoside	Asiaticoside	Indonesia	155

*Centella asiatica* has been used in Ayurvedic medicine in India for almost 2000 years with significant use of its neuropharmacological properties.^6^ In Ayurveda, it has been recognized as one of the main herbs for revitalizing the nerves and brain cells and has been administered extensively in treating disorders like depression. *C. asiatica* parts were considered useful in the diseases of the skin, nervous system and blood. Although the leaf was initially given importance in the traditional pharmacopoeia of India, many modern investigators have advocated the use of entire plant, root, twigs, leaves and seeds in medicine.^7^ The use of this herb can also be traced back to China and other Southeast Asian countries where it was used for fever, skin conditions and treating inflammation‐related diseases.^8^, ^9^ In Assam, this herb has been traditionally used as an antimicrobial against gut infections and other gut ailments.^10^ During the middle of the twentieth century, *C. asiatica* and its alcohol extracts were reported to have shown positive results in the treatment of leprosy in Western medicine.^11^

In the last few years, a variety of medicinal herbs and bioactive compounds extracted from plant sources have exhibited therapeutic properties that are beingp currently investigated extensively for clinical use. A majority of these are native to South Asia, South‐East Asia and Africa. Extracts from plant species including members of the Acanthaceae family (*Andrographis paniculata* ^12^ and *Lepidagathis hyalina* ^13^), the Gynura (Compositae) genus,^14^ *Syzygium fruticosum*,^15^ *Psychotria calocarp*,^16^ *Boerhavia diffusa* ^17^ *and Molineria capitulate* ^18^ among others have shown a range of ethnopharmacological benefits in treating different ailments. The ‘superfood’ photosynthetic bacteria *Spirulina platensis* ^19^ and leaves from the medicinal herb *Ophiorrhiza rugosa* ^20^ are examples of natural anti‐inflammatory agents that have been recently seen to demonstrate pain‐suppressing (antinociceptive) activities. The phytochemical class of furanocoumarins has proved particularly beneficial in the promotion of anti‐cancer pathways in leukaemia, glioma, breast, lung, renal, liver, colon, cervical, ovarian and prostate tumours by regulating a number of cancer‐associated cell signalling cascades.^21^ Similarly, coumarin derivatives have shown strong anti‐cancer activity, particularly in prostate cancer, renal cell carcinoma and leukaemia,^22^ whereas bioactive compounds such as flavonoids^23^ and metabolites of cruciferous vegetables^24^ of the Brassicaceae family have shown promising results in inducing anti‐tumour effects in colorectal cancer cell lines. Other plant compounds including *Aglaonema hookerianum* ^25^ and the monoterpenoid alcohol terpineol^26^ are also seen to display neuroprotective properties and are being studied for their biological action in ameliorating depression and anxiety. To improve the bioavailability of several therapeutic agents extracted from plant sources, nanocarrier systems are currently being investigated for medical use,^27^ and advancements in such drug delivery approaches can also dramatically benefit the clinical applications of natural compounds like these.

Presently, various aqueous and alcohol extracts of *C. asiatica* are utilized to ameliorate a broad range of diseases and disorders, with abundant in vitro and animal studies supporting the therapeutic actions of asiaticoside and madecassoside in particular.

**FIGURE 2:**
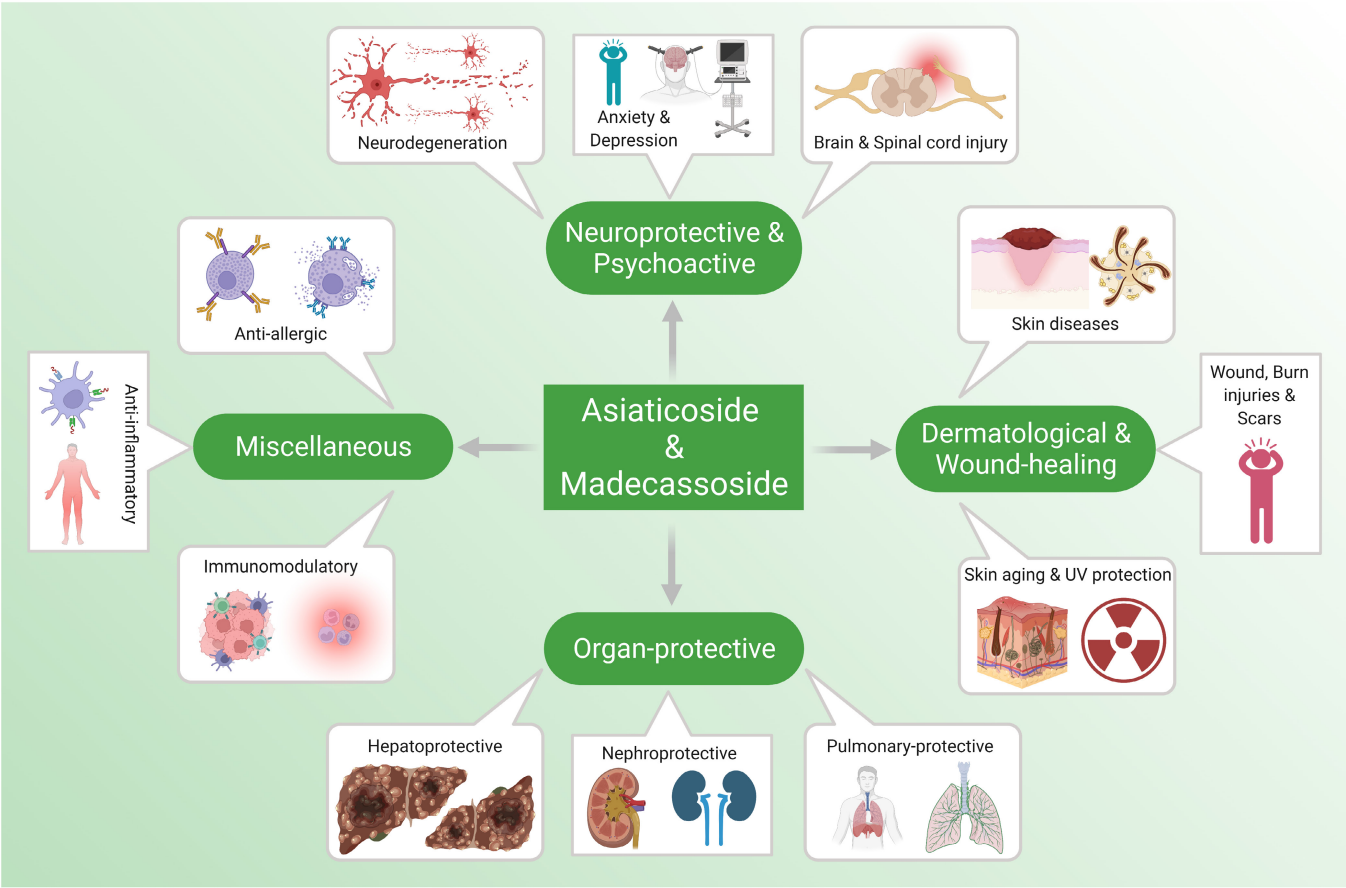
Various pharmacological attributes of asiaticoside and madecassoside

Among the many activities of these two phytochemicals of interest, the most widely used ones are neuroprotective, wound‐healing and skin‐protective properties. Asiaticoside can alleviate neuronal damage,^28^ shows anti‐anxiety effects^29^ and can behave as an anti‐depressant‐like agent.^30^ Madecassoside and asiaticoside are implicated in the treatment of hypoxic–ischaemic injuries in the brain^31^, ^32^ and are incorporated into the treatment of various neurodegenerative disorders like Alzheimer's and Parkinson's diseases.^33^, ^34^ Furthermore, these compounds have collagen‐stimulating, hydrating, scar and burn‐healing properties,^35^, ^36^ resulting in their popularity in both clinical and cosmetic contexts. These triterpene saponins are also confirmed to play a significant role in improving organ injuries or damage, repressing inflammation and oxidative stress, protecting against bacterial, fungal and parasitic infections,^10^, ^37^, ^38^ exhibiting chemotherapeutic effects,^39^ as well as regulating various immune responses. Hence, they are strong candidates for natural‐based pharmacology. This review describes the broad range of pharmacological benefits of these phytocompounds, illustrating their potential in clinical applications for a number of diseases and disorders (Figure 2).

## 2 NEUROPROTECTIVE AND PSYCHOACTIVE PROPERTIES

Neurological disorders accounted for approximately 9·0 million of total worldwide deaths in 2016 (including people with disabilities). The steepening global burden of these diseases with ageing populations poses an increasing challenge in treatment and rehabilitation, urging the need for effective developments in medication and therapeutic strategies.^40^ The most extensively studied medicinal benefits of asiaticoside and madecassoside are their neurotherapeutic properties. These two active compounds extracted from *C. asiatica* are seen to shield the brain against neurodegenerative disorders and cognitive deficits, enhance memory and learning, alleviate symptoms of depression and anxiety and exhibit overall protection over the central nervous system.

To be clinically effective, administered neurotherapeutic drugs must be able to cross the blood–brain barrier (BBB). The blood–brain barrier (BBB) is a continuous endothelial membrane within the brain micro‐vessels where the brain endothelial cells are sealed by tight junctions, characterizing low paracellular and transcellular permeability.^41^ It behaves as a complex, regulatory interface that is interlinked with the rest of the Central Nervous System (CNS) and peripheral tissues; it dynamically adapts to the needs of the CNS, responds to physiological changes^41^ and controls the exchange between blood and the cerebral tissue to maintain brain homeostasis as well as to block the entry of toxic substances.^42^, ^43^, ^44^

The disruption of the BBB is involved in the pathophysiology of various neurological diseases such as Alzheimer's disease (AD),^45^ and traumatic brain and spinal cord injuries.^46^ Treating CNS diseases requires therapeutics that can cross and sufficiently interact with the BBB. Many pharmaceuticals are rendered ineffective against neurological disorders because they fail to penetrate the BBB, not because they lack insufficient potency.^47^ This limits the medical benefits of several types of CNS drug delivery.^48^, ^49^ A study by Hanapi, Nur Aziah et al. investigated the extent of BBB permeation by *C. asiatica* compounds using primary porcine brain endothelial cells (PBECs) as a model. The experiment demonstrated the remarkably high capability of the tested phytocompounds in crossing the BBB, with asiaticoside showing the highest permeability, followed by madecassoside. Notably, the compounds also showed higher permeability coefficient values than donepezil, a drug commonly used for AD.^43^

The complexity of the BBB membrane provides possible routes for disease manifestation as well as for a wide range of drug‐delivery approaches. The disruption of the BBB has been repeatedly attempted as a strategy for drug administration. However, techniques that disrupt an intact BBB for delivering a targeted drug require meticulous monitoring, as this process can simultaneously allow the entry of toxic circulating substances and microbial pathogens into the cerebral tissue, which is normally shielded by the BBB.^42^ Other consequences of BBB damage and loss of integrity include reduced cerebral blood flow, impaired hemodynamic response, dysregulated molecular and ionic flux, impaired transporter function and leakage of plasma proteins, which can all result in multiple paths of complications involving neuronal dysfunction, neuroinflammation and neurodegeneration.^50^ In the same study using primary porcine brain endothelial cells, asiaticoside and madecassoside were proved to be efficient in crossing the BBB at a high rate without exerting any toxic effects, and while preserving the BBB tight junction integrity, making them desirable neurotherapeutics.

### 2.1 Neurodegenerative diseases

Progressive, debilitating conditions which bring about the eventual degradation of neuronal cells are classified as neurodegenerative diseases. The most widely studied neuroprotective applications of asiaticoside and madecassoside are their immunomodulatory effects on AD and Parkinson's Disease (PD). Both diseases are progressive and involve neuronal loss and damage to neuronal circuits. AD pathogenesis is mainly identified by extracellular aggregates of amyloid β (Aβ) peptides and the formation of intracellular neurofibrillary tangles.^51^ Most patients with AD are diagnosed at a stage where neuropathologic lesions have already developed, resulting in cognitive deterioration and memory difficulties.^52^ Individuals affected by PD suffer major degeneration to the dopamine‐transmitting neuronal network in their midbrain, causing serious harm to the motor cortical regions of the brain before they are met with debilitating movement abnormalities such as gait impairment, rest tremor and poor coordination.^53^

Age‐related alterations in the brain microenvironment gradually led to increased BBB permeability and leakiness, neuronal degeneration, production of reactive oxygen species (ROS) and inflammation.^54^, ^55^, ^56^ Oxidative stress is triggered by a state of imbalance between ROS (chemically reactive species such as hydrogen peroxide (H_2_O_2_) and hydroxyl radicals (OH^·^)) and antioxidants. Excess ROS accumulation or reduced antioxidant levels can directly hamper neuronal synaptic activity and neurotransmission.^8^, ^57^ Oxidative stress is associated with a number of inflammatory and degenerative pathologies and is a significant biomarker common to the pathophysiology of both AD and PD.^55^, ^58^

The triterpene compounds obtained from aqueous or ethanolic extracts of *C. asiatica*, which majorly include asiatic acid, asiaticoside and madecassoside, are proven to exhibit high levels of free radical‐scavenging capacity and strong reducing potential. Aggregated Aβ‐induced ROS generation and subsequent neurotoxicity are critical to the pathogenesis of AD; administration of *C. asiatica* extracts into PC12 pheochromocytoma cells and human IMR32 neuroblastoma cells bearing Aβ‐induced oxidative stress have resulted in overall antioxidant activity. The mechanism of this action is via modulation of the antioxidant defence system and direct elimination of uncontrolled ROS production by *C. asiatica* extracts.^59^ Studies indicate that the antioxidant activity of *C. asiatica* extracts is largely attributed to its active triterpene constituents, especially asiaticoside and asiatic acid. An experiment checking for their activity against Parkinson's used rodent models treated with MPTP (a neurotoxin that destroys dopaminergic neurons and induces ROS formation) to demonstrate the action of *C. asiatica* extracts in enhancing brain antioxidants, decreasing lipid peroxidation and providing neuroprotection against MPTP toxicity.^60^ In the aforementioned study by Hanapi et al.,^43^ madecassoside, in particular, was observed to protect PBECs against H_2_O_2_‐induced oxidative stress. Similar protective action of madecassoside has been identified in endothelial cells, as detected in H_2_O_2_‐treated human umbilical vein endothelial cells (HUVECs). Madecassoside treatment reduced the effects of oxidative stress on the endothelial cells, primarily by prevention of lipid peroxidation and inhibition of pro‐apoptotic factors which were otherwise upregulated as a response to elevated ROS levels. In addition, the compound preserved brain mitochondrial function.

Chronic neuroinflammation is another distinct biomarker observed at the early stages of neurodegenerative disease. Increasing concentrations of pro‐inflammatory cytokines aggravate both AD and PD pathogenesis by stimulating neurotoxic plaque formation and Aβ depositions in AD patients, as well as causing neurodegenerative lesions in PD. These conditions lead to synaptic dysfunction and gradual neuronal death.^8^ Hafiz et al. have characterized the therapeutic potential of raw extract of *C. asiatica* (RECA) by simultaneously demonstrating the anti‐inflammatory as well as the antioxidant activities of RECA in LPS‐stimulated microglial cells. Exposure of neurons and oligodendrocytes to LPS subjects them to elevated inflammation (due to potent pro‐inflammatory cytokines including NO, PGE2 and TNF‐α) and overwhelming oxidative stress as a result of perpetual overactivation of the microglia. RECA displayed strong anti‐inflammatory activity by suppressing the inflammatory mediators in a concentration‐dependent manner; however, despite the highest triterpene constituent of RECA being characterized to be madecassoside, followed by asiaticoside, the anti‐inflammatory action seen in this study was instead attributed to the comparatively lower madecassic acid content. At the same time, the high proportions of madecassoside in RECA were proven to be responsible for restoring antioxidant activity in the microglia as well as suppressing intracellular ROS generation, hence reversing the LPS‐induced oxidative damage. Similar properties were observed in vivo using LPS‐treated Sprague Dawley rats, where administration of RECA significantly prohibited or restored oxidative stress and neuroinflammation. In addition, RECA exhibited curative and protective properties against AD by attenuating acetylcholinesterase activity both in vitro as well as in the LPS‐injected rodent models of AD.^61^

Aβ_1–42_ deposition is retained in the brains of AD patients and is involved in the initiation of fibrillar deposition of amyloid‐beta peptides in the brain. A study by Hossain, Shahdat et al. demonstrated the amyloidogenesis‐inhibitory action of asiaticoside using fluorescence correlation spectroscopy, where asiaticoside was seen to block Aβ_1–42_ fibrillation in the early stages. In addition, a previous study by the same team has also accounted for the role of madecassoside in inhibiting Aβ_1–42_ fibrillation as well as improving memory loss of AD model rats.^33^

Another prominent manifestation in AD patients has reduced levels of the neurotransmitter acetylcholine (ACh) in the brain cortex and hippocampus. Thus, a favourable therapeutic target for AD would be the inhibition of acetylcholinesterase enzyme (AChE) which is responsible for the hydrolytic breakdown of ACh. Orhan et al. demonstrated that ethanolic extract of *C. asiatica* (with 10.78% content of asiaticoside and madecassoside) exhibited roughly 50% inhibition against AChE, indicating that this strong neuroprotective activity can be attributed to the high concentrations of triterpene saponins in the extract.^62^

Asiaticoside‐treated MPTP rat models of Parkinsonism have shown preservation of dopaminergic neurons by inhibiting MPTP‐induced neurotoxicity and also maintaining the metabolic balance of dopamine, protecting against locomotor dysfunction, preventing oxidative damage and upregulation of Bcl‐2 expression while simultaneously reducing levels of pro‐apoptotic protein Bax. Most significantly, asiaticoside tackled oxidative stress by reducing the production of malondialdehyde (MDA) levels which induce oxidative damage to proteins and lipids involved in PD, and also increased the expression of the potent antioxidant glutathione (GSH). Furthermore, the high Bcl‐2/Bax ratio accounts for reduced ROS generation and higher GSH‐stabilized antioxidant activity via the action of Bcl‐2, thus contributing to enhanced resistance against MPTP‐induced Parkinsonism.^63^ Similar mechanism of action had been observed in an earlier study using MPTP‐treated mice where asiaticoside administration prevented oxidative damage to the neurons and protected against Parkinson's toxicity specifically by blocking Bax‐mediated neuronal death and promoting Bcl‐2 expression.^64^

Madecassoside also exhibits similar properties, as seen in another study where the compound prevented early signs of Parkinsonism in MPTP‐treated rats by reversing the depletion of dopamine, upregulating antioxidant activity and increasing the Bcl‐2/Bax ratio.^34^

### 2.2 Brain and spinal cord injury

The majority of strokes are due to cerebral ischaemia, which is one of the leading causes of disability and death. Acute ischaemic strokes are considered thrombo‐inflammatory diseases and are primarily treated by rapid re‐establishment of blood flow (recanalization) of occluded cranial vessels. However, infarcts can grow despite successful recanalization, leading to serious damage known as cerebral ischaemia/reperfusion injury or CIRI.^65^ Zhang et al. evaluated the effects of asiaticoside in vitro as well as in vivo using rat models for cerebral ischaemia/reperfusion injury. Asiaticoside successfully reversed nervous function injury, brain oedema, cell apoptosis, infarct size, apoptosis‐related protein expressions, inflammation and oxidative stress, and also increased cell survival by blocking the NOD2/MAPK/NF‐κB signalling pathway.^66^ Madecassoside exerts robust protection against cerebral ischaemia–reperfusion (I/R) injury as seen in a study by Luo et al. Madecassoside shows similar mechanistic actions of anti‐oxidative, anti‐inflammatory and anti‐apoptotic activity against I/R injury by attenuating microglia‐mediated neuroinflammation via the inhibition of the TLR4/MyD88/NF‐κB signalling pathway.^67^ Other studies verify the role of asiaticoside and madecassoside in checking cerebral ischaemic injury, particularly hypoxic‐ischaemia. Asiaticoside has previously shown a protective effect on ischaemia‐hypoxia neural cells in‐vitro and promotes ischaemia‐hypoxia nerve cell survival rate by upregulating anti‐apoptotic protein Bcl‐2, reducing cell apoptosis by suppressing pro‐apoptotic factors Bax and caspase‐3. Asiaticoside can further inhibit lactate dehydrogenase release by ischaemia‐hypoxic cells and inhibit membrane lipid peroxidation, thereby preventing damage to neuronal membranes and blocking ischaemia‐associated neuron necrosis.^32^ Hypoxia stimulates alterations in calcium ion concentration which in turn stimulates the mitochondrial apoptotic pathway, hence activating the production of reactive oxygen species, resulting in hypoxic–ischaemic‐induced brain damage (HIBD). Asiaticoside has been proven useful in preventing HIBD‐induced apoptosis, lowering the production of pro‐inflammatory cytokines and repairing brain neuron injury and oxidative damage in a dose‐dependent manner via the TLR4/NF‐κB/STAT3 pathway. This is a significant development in the clinical treatment of neonatal hypoxic–ischemic encephalopathy (HIE) which is a serious risk for neonatal death.^68^

Spinal cord injuries (SCI) are traumatic and frequently cause neurological dysfunction. SCIs can lead to changes in brain structure and brain function by inducing nerve damage as well as causing long‐term complications like paralysis and neuropathic pain.^69^ A key event in mediating cellular response to spinal cord injury is inflammation. Asiaticoside treatment has proven to significantly reduce the expression of the pro‐inflammatory marker TNF‐α in spinal cord tissue, as revealed by low TNF‐α levels in the serum of SCI‐induced rats in a reported study. Moreover, the triterpene functioned by blocking the apoptosis of spinal cord neurons and enhancing neuronal survival, thereby promoting recovery.^70^ Luo et al. investigated the beneficial actions of asiaticoside in SCI rat models. The results attested to the neuroprotective properties of asiaticoside in attenuating SCI which prevented oxidative damage, nitric oxide activity and the production of pro‐inflammatory cytokines. Furthermore, asiaticoside also inactivated the p38‐MAPK signalling pathway, which when expressed leads to the destruction of the blood–spinal cord barrier.^71^ Hence asiaticoside in particular can be considered a major therapeutic compound in treating SCI, although the effects of asiaticoside on neurological pain remain to be fully elucidated.

### 2.3 Anxiety and depression

Generalized anxiety disorder (GAD) is a highly disabling mental health condition and is frequently aggravated further due to other psychiatric disorders, such as major depressive disorder (MDD), panic disorder, somatic symptom disorders and personality disorders. The development of anxiety disorders includes an interaction of psychosocial factors such as childhood adversity, stress or trauma, along with genetic vulnerability, which manifests into neurobiological and neuropsychological dysfunctions. GAD often precedes depression, another prevalent mental condition that depletes the quality of life and is associated with sustained stress, marked distress and medical comorbidity. Several neurobiological markers of these conditions have been established and continue to be reviewed, and the commonly used forms of treatment at present are psychological therapy (eg: cognitive behavioural therapy), pharmacotherapy, or a combination of both. However, GAD and depression, along with associated mental disorders, remain largely marginalized and are frequently overlooked and undertreated in primary care, leading to poor treatment outcomes. Moreover, the adverse side‐effects of antidepressants and anti‐anxiety (anxiolytic) medications remain to be fully illustrated.^72^, ^73^, ^74^ The lack of fully effective therapeutic approaches towards GAD and depression facilitates the need for alternative avenues of treatment, including those of natural origin, that can be achieved by incorporating medicinal plant extracts into primary care routines.

A 70% hydroethanolic extract of *C. asiatica* has previously shown promising anxiolytic properties in a clinical study where it attenuated anxiety‐related disorders, stress phenomenon and correlated depression in GAD patients.^75^ Further examination of *C. asiatica* as an anxiolytic agent proves its efficiency in ameliorating the pathological stress manifested due to anxiety. Wanasuntronwong, Aree et al. used a standardized extract of *C. asiatica* (ECa 233) to evaluate its anti‐anxiety effects on acutely stressed as well as chronically stressed mice. The therapeutic action of Eca 233 was found comparable to a well‐known anxiolytic drug, diazepam. With specific respect to the two major triterpenoids, asiaticoside and madecassoside, there was significant relief from anxiety‐induced stress, however, there was no such distinct effect on other well‐defined physiological markers of chronic stress like body weight or serum corticosterone.^76^

A recent study also implicates the significance of asiaticoside, among other candidate drugs, as an emerging anti‐depressant.^77^

A potential neurobiological target in depression is inflammation.^78^ Depression‐associated neuroinflammation activates pro‐inflammatory cytokines and thereby increases neuronal susceptibility to damage, affects serotonin synthesis and metabolism, alters neuronal apoptosis, and impairs neurogenesis and neuroplasticity. Inflammatory cytokines also regulate monoamine neurotransmitter metabolism by inhibiting neurotransmitter synthesis (serotonin, norepinephrine and dopamine) with studies suggesting that cytokines are involved in serotonin depletion in the CNS.^79^ A recent study highlights the anti‐inflammatory properties of asiaticoside in treating depression in chronic unpredictable mild stress mouse models; asiaticoside administration can reverse depressive‐like behavioural patterns, increase the levels of monoamine neurotransmitters, and inhibit hippocampal inflammation. The observed antidepressant‐like effect is believed to occur by regulation of the cAMP/PKA signalling pathway to inhibit NF‐κB‐ and NLRP3‐mediated inflammation.^30^

## 3 DERMATOLOGICAL AND WOUND‐HEALING PROPERTIES

Several natural compounds are utilized in treating skin defects and wounds, as a topical medicine for acne or scars, or for cosmetic purposes. Different extracts (TECA, TTFCA, ethanolic and methanolic) of *C. asiatica* as well as its individual triterpene constituents asiaticoside, madecassoside, asiatic and madecassic acid, have shown significant applications in the treatment of dermatoses and skin lesions such as excoriations, burn injuries, cutaneous scars (hypertrophic scars and keloid scars), eczema, as well as in the healing process of skin wounds.^80^ Asiaticoside is also a popular phytocompound currently used in anti‐ageing agents.

### 3.1 Skin diseases

Atopic dermatitis (or atopic eczema) is one of the most common allergic inflammatory skin diseases and is caused by abnormal immune responses associated with skin barrier dysfunction.^81^

The pharmacological effects of *C. asiatica* on 2,4‐dinitrochlorobenzene (DNCB)‐induced skin inflammation has been tested using in vitro and in vivo models of atopic dermatitis, and the results illustrated strong protective activity of *C. asiatica* extract whereby it inhibited pro‐inflammatory cytokines to effectively suppress dermatitis symptoms, most significantly the reduced infiltration of immune cells into dermal tissue. This immunosuppressive action may be attributed to the anti‐allergic and anti‐inflammatory properties of its constituent compounds.^82^

Madecassoside has been implicated as a promising treatment for the skin condition vitiligo, an acquired pigmentary disorder of the skin and mucous membranes that is characterized by chronic and progressive loss of melanocytes from the epidermis and the follicular reservoir. Evidence shows that oxidative stress caused by H_2_O_2_ is a major contributor to the onset and progression of vitiligo.^83^ An experimental study to test the effects of *C. asiatica* on oxidative stress in human melanocytes demonstrated that madecassoside can protect the mitochondrial structure against ROS overproduction and attenuate the overall oxidative damage in melanocytes via the activation of autophagy.^84^

Another prevalent skin disorder of concern is acne. Hydration and anti‐inflammation are considered crucial steps in maintaining skin homeostasis and barrier function; however, these can be compromised by the development of acne, a chronic dermatologic disorder characterized by major pathophysiological factors of increased seborrhea, hyperkeratinization of the pilosebaceous unit and inflammation caused by the skin commensal *Propionibacterium acnes*.^85^ Madecassoside is confirmed to significantly protect the skin against acne inflammation by inhibiting the production of pro‐inflammatory cytokines, IL‐1β and TLR2, released by *P. acnes* in *P. acnes*‐stimulated THP‐1 human monocytic cells. Moreover, madecassoside can notably enhance skin hydration and moisturization both in vitro and in vivo, proving to be both medically and cosmetically beneficial.^36^

### 3.2 Skin ageing and UV protection

Cutaneous ageing is influenced by both intrinsic factors (chronologic ageing) and environmental damage, majorly UV radiations from the sun (photoaging).^86^ The effects of skin ageing (sagging, wrinkle formation) are most prominent in the superficial dermis and epidermis. Topical treatments using madecassoside^87^ and asiaticoside.^88^, ^89^ show improvements in hyperpigmentation,^88^, ^90^ photoaging skin, cellulite and striae^91^ and periocular wrinkles.^87^, ^89^, ^92^

### 3.3 Wounds, burn injuries, and scars

Wound healing is a dynamic process and the healing process starts immediately after a skin injury, taking months to complete.^93^ Asiaticoside and madecassoside show unique pharmacological properties of wound healing and have been extensively tested in vitro and in vivo, although there are few verified studies on humans. The wound‐healing mechanism of standardized *C. asiatica* extracts ECa 233 has been assessed by investigating its effects on the migration of a human keratinocyte cell line (HaCaT) using scratch wound‐healing assay. Keratinocyte migration was significantly enhanced by ECa 233 in a concentration‐ and time‐dependent manner. The observed wound‐healing activity of ECa 233 occurs via the activation of FAK, Akt and MAPK‐dependant signalling pathways.^94^ Other controlled studies have further confirmed the role of asiaticoside and madecassoside in enhancing wound healing and diminishing hypertrophic and keloid scars.^95^ Keloid is an excessive dermal scar occurring in response to skin injuries. While hypertrophic scars do not spread beyond the site of injury, keloid scars extend beyond the original wound edges and invade the adjacent normal dermis due to extensive production of extracellular matrix, especially collagen, caused by overproduction of cytokines and growth factors. Keloids are invasive in nature and characterized by the migratory behaviour of overactive fibroblasts.^93^ They are associated with signalling pathways like transforming growth factor‐beta 1 (TGF‐β1), mitogen‐activated protein kinase (MAPK) and insulin‐like growth factor‐I (IGF‐I), which can be regulated by phytochemicals like asiaticoside and madecassoside.^95^ Madecassoside has been seen to suppress the migration of keloid‐derived fibroblasts (KFs) as observed in madecassoside‐treated fibroblasts taken from human earlobe keloids. To migrate, a cell must develop morphological polarity, continuously protrude a single lamellipodium and then polarize in the direction of migration through organized actin polymerization. Madecassoside can directly reduce the cytoskeletal protein actin expression of KFs and its mechanism of action is by inhibiting the activity of some (not all) of the several intracellular molecules that are responsible for abnormal keloid migration, including the significant, concentration‐dependent reduction of phosphorylated Akt, PI3K and p38 of KFs and the repression of actin‐depolymerization‐associated p‐cofilin/cofilin.^96^

The overexpression of growth differentiation factor‐9 (GDF‐9) in keloids enhances the proliferation, migration and invasion of keloid fibroblasts due to the phosphorylation of Smad 2/3 proteins through activation of the MAPK pathway. Asiaticoside is capable of inhibiting this invasive growth of KFs by inhibiting the GDF‐9/MAPK/Smad pathway.^97^

Despite available treatment options for keloid scars such as a combination of medical and surgical therapies, the reoccurrence rate is still high.^93^ This prompts clinical treatments to explore natural therapeutic options as well, taking into consideration the efficacy of asiaticoside and madecassoside in successfully treating scars. Interestingly, the effects of the mentioned phytochemicals on reducing fibroblast migration do not extend to repressing collagen production, which is also over‐expressed in keloid scar formation. Instead, asiaticoside and madecassoside prove cosmetically useful by inducing the production of collagen and thus preventing skin‐ageing, which is caused as a result of low Type 1 collagen secretion in the dermis. Asiaticoside can induce type I collagen synthesis via the activation of the TGF‐β receptor I kinase‐independent Smad signalling.^98^ Both asiaticoside and madecassoside have been observed to stimulate collagen production in the context of burn wound healing.^35^

Burn wound healing is a complex process involving inflammation, re‐epithelialization, granulation, neovascularization and wound contraction, and it requires the activity of several biochemicals including antioxidants and cytokines. Extensive in‐vitro and in‐vivo studies indicate that *C. asiatica* is one of the best medicinal plants for burn wound‐healing activity, with asiaticoside and madecassoside proving especially effective.^99^ Madecassoside has significant wound‐healing activity based on several mechanisms including anti‐inflammatory and antioxidative activity, collagen synthesis and angiogenesis, contributing greatly to the use of *C. asiatica* herbs as a favourable source for treating burn injuries.^100^ Asiaticoside also enhances burn wound healing through the promotion of angiogenesis during skin wound repair.^101^ Asiaticoside and madecassoside are recognized as the main active constituents involved in healing burns, whereby orally administrated madecassoside shows significantly higher efficiency in the synthesis of procollagen, wound‐healing speed and wound‐healing pattern, as observed in vitro in primary skin fibroblasts and in vivo in mice that have sustained burn injuries.^35^ Furthermore, the wound‐healing activities of sequential hexane, ethyl acetate, methanol and water extracts of *C. asiatica* have been identified in incision and partial‐thickness burn wound models in rats. All extracts, including those containing asiaticoside, madecassoside and asiatic acid, showed remarkable enhancement of burn wound healing with fully developed epithelialization and keratinization observed in all the extract‐treated groups. However, asiatic acid was seen to perform more efficiently compared to our phytocompounds of interest.^102^

## 4 OTHER ORGAN‐PROTECTIVE EFFECTS OF ASIATICOSIDE AND MADECASSOSIDE

### 4.1 Hepatoprotective properties

Intararuchikul, Thidarat et al. identified that supplementation of the standardized extract of *C. asiatica* (ECa233) protects rat liver against rotenone toxicity (a natural pesticide), and ECa233 administration prior to rotenone exposure inhibits lipid peroxidation in liver tissue and also enhances the expression of catalase endogenous antioxidant enzyme. This hepatoprotective activity can be attributed to the antioxidant properties of the constituent triterpenoids in the herb.^103^

An earlier study was carried out on the effect of asiaticoside, madecassoside and asiatic acid on cyclophosphamide (CYP)‐induced hepatotoxicity and immunosuppression in rats. Upon administration of the triterpene saponins, the relative weights of immune organs were restored to normal in the CYP‐treated rats, the production of inflammatory cytokines was suppressed, and the previously low levels of antioxidants were restored. Overall, the saponins can protect against hepatic damage against immune‐mediated liver disease and also prevent multiorgan injury.^104^

Asiaticoside and madecassoside have shown significant protection against lipopolysaccharide (LPS)/D‐galactosamine (D‐GaIN)‐induced acute liver injury in two separate studies. Asiaticoside presented anti‐inflammatory effects and dose‐dependently reduced elevated aminotransferases, hepatocytes apoptosis and caspase‐3, as well as improved mortality and liver pathological injury. Furthermore, asiaticoside inhibited the expression of TNF‐alpha and MAPKs to protect against hepatic damage.^105^ Madecassoside exhibited both antioxidative and anti‐inflammatory effects in LPS/D‐GalN‐induced hepatic damage in mice. Madecassoside attenuated liver injury by protecting liver function, suppressing the production of inflammatory cytokines (TNF‐α, IL‐1β and IL‐6), restoring/enhancing antioxidant enzyme activity and suppressing LPS‐stimulated proteins.^106^ Thus asiaticoside and madecassoside are promising hepatoprotective agents for acute liver injury or liver failure.

Drug‐induced liver injury and acute liver failure (ALF) are often caused by accidental overdose and adverse side effects of the drug N‐acetyl‐p‐aminophenol (acetaminophen, APAP) which is a popular analgesic and antipyretic agent; excessive APAP results in oxidative stress in liver tissues due to increase in MDA content and reduction in GSH levels, as well as inflammatory cell infiltration and overexpression of pro‐inflammatory cytokines (such as TNF‐α and IL‐1β), ultimately leading to hepatoxicity. The observed GSH depletion, MDA overproduction and overexpressed inflammatory markers caused by APAP have been seen to be reversed by CA‐HE50 treatment. The antioxidant and anti‐inflammatory actions were identified to be due to the asiaticoside content present in the extract. Hence, asiaticoside is an effective phytochemical in protecting against APAP‐induced liver injury.^107^

### 4.2 Pulmonary‐protective properties

Pulmonary arteries have thinner walls and less vascular smooth muscles than systemic circulation, and one of the factors controlling pulmonary blood flow between the lungs and other organs is the vascular structure. Low oxygenation or hypoxia in tissues causes pulmonary vasoconstriction and if it persists long term, the contraction is followed by remodelling of the vasculature, resulting in pulmonary hypertension.^108^ Pulmonary hypertension is associated with chronic lung diseases such as chronic obstructive pulmonary disease (COPD), cystic fibrosis and bronchopulmonary dysplasia and is linked with reduced functioning and poor patient outcomes.^109^ Asiaticoside has been found to play a significant role in ameliorating hypoxia‐induced pulmonary hypertension (PH).

Wang, Xiaobing et al. demonstrated in an earlier study that asiaticoside can attenuate the development of PH in hypoxia‐induced PH rat models by attenuating pulmonary cardiovascular remodelling and preventing right ventricular hypertrophy. These results were concluded to be most likely mediated by the action of asiaticoside in blocking the hypoxia‐induced overactivity of the transforming growth factor (TGF)‐β1/SMAD family member (SMAD) 2/3 signalling pathway as well as inhibiting the aberrant proliferation and migration of pulmonary arterial smooth muscle cells (PASMCs) which is a hallmark of vascular remodelling, by inducing their apoptosis.^110^ Research indicates that abnormal endothelial cells (ECs) apoptosis and ECs dysfunction are involved in the initiation and progression of PH. The same team conducted another study recently to further explore the effects of asiaticoside on hypoxic pulmonary hypertension in rats and in human pulmonary artery endothelial cells (HPAECs) but with a special focus on the influence on endothelial cell function. Hypoxia interferes with NO production causing irregular morphology and dysfunction of endothelial cells experiencing PH; asiaticoside can activate nitric oxide (NO) production by enhancing the phosphorylation of serine/threonine‐specific protein kinase/eNOS, thus preventing ECs from hypoxic PH‐induced apoptosis. Asiaticoside was also found to restore misadjusted levels of vascular mediators, preserve EC morphology and function, and protect against EC cell injury. In addition, asiaticoside was confirmed to regulate the PI3K/Akt signalling pathway both in vivo and in vitro to promote cell survival and viability.^111^

Asiaticoside also protects against lipopolysaccharide (LPS)‐induced acute lung injury (ALI); the major feature of ALI is uncontrolled inflammatory responses in the lung and the NF‐κB pathway is linked to the cytokines production and regulation of inflammatory responses.^112^ Asiaticoside has been studied to dose‐dependently reduce LPS‐induced pulmonary inflammation by inhibiting inflammatory infiltration, histopathological changes, cytokine production and LPS‐induced pulmonary oedema via down‐regulation of the NF‐κB signalling pathway.^113^ Similarly, madecassoside exerts anti‐inflammatory action by downregulating the TLR4/NF‐κB signalling pathway in LPS‐induced acute lung injury, and also protects the lungs by repairing LPS‐induced pathological damages, lung oedema and alveolar epithelium integrity.^114^ Another serious lung disease is idiopathic pulmonary fibrosis (IPF) which is a chronic, progressive interstitial lung disease characterized by the aberrant proliferation of fibroblasts, excessive extracellular matrix (ECM) and chronic inflammation with poor prognosis; asiaticoside is known to attenuate bleomycin‐induced pulmonary fibrosis by activating cAMP and Rap1 signalling pathways, thus inhibiting inflammation and fibrosis in the lungs.^115^ Madecassoside can also treat bleomycin‐induced pulmonary fibrosis by preventing the deposition of extracellular matrix, achieved mainly through attenuating inflammation and oxidative stress in early stages of pulmonary fibrosis as well as repressing collagen overproduction.^116^

Bronchopulmonary dysplasia (BPD) is another common pulmonary disease seen in premature infants with no effective therapy. BPD‐associated pathological changes can cause hyperoxia‐ induced lung injury (HILI) in immature lung tissue, which can be markedly attenuated by asiaticoside via anti‐inflammation and anti‐apoptosis pathways in vitro and in vivo.^117^

### 4.3 Nephroprotective properties

Asiaticoside and madecassoside offer protection against renal diseases as well. Kidney inflammation initiates renal fibrosis, which results in progressive renal dysfunction and leads to chronic kidney disease (CKD) and eventually end‐stage renal disease.^118^ Asiaticoside protects against renal fibrosis in vitro and in vivo as observed in a recent study.^119^ Asiaticoside also has a therapeutic role against nephropathy; nephropathy is a microvascular complication of diabetes mellitus that often leads to terminal renal failure and is generally characterized by structural and functional abnormalities in podocytes including podocyte hypertrophy, effacement and apoptosis.^120^ Nephropathy can be inhibited by preventing podocyte injury and by ameliorating proteinuria. Asiaticoside treatment can mitigate the kidney histological damages induced by nephropathy. Furthermore, asiaticoside is seen to reduce urine protein excretion (proteinuria) in adriamycin‐induced nephropathy rats. Asiaticoside is also observed to increase the expression of synaptopodin and decrease desmin expression in rat models, which restores the expression of cytoskeletal proteins in damaged podocytes and subsequently restores normal renal morphology. Asiaticoside also helps repair the glomerular filtration barrier of the kidneys which gets compromised in nephropathic progression.^121^ In another study using a *C. asiatica* extract, one of the major components was asiaticoside and it demonstrated several pharmacological effects in diabetic rats such as restoring the activities of kidney enzymes involved in glucose and amino acid oxidation in diabetes and protecting diabetic tissues from stress via antioxidant mechanisms to protect against diabetic kidney disease.^122^ Asiaticoside is the major constituent of the formula Compound Centella, which has proved effective in lowering the urine protein/creatinine ratio in diabetic rats and can improve the renal pathology of diabetic kidney disease by regulating oxidative stress signalling.^123^ Madecassoside exhibits nephroprotective properties by protecting against nephrotoxicity; the chemotherapeutic drug doxorubicin (DOX) results in multi‐organ injury including nephrotoxicity, during which it induces the apoptosis of Human Proximal Tubule Cells HK‐2 cells. This DOX‐induced cytotoxicity can be suppressed by treating with madecassoside, as it inhibits DOX‐associated apoptosis and inflammation in HK‐2 cells and can be considered for optimizing the DOX chemotherapeutic approach.^124^

## 5 MISCELLANEOUS ACTIVITIES

Asiaticoside and madecassoside offer numerous other medicinal benefits in treating cardiac, pancreatic and colon diseases. Various extracts and compounds isolated from *C. asiatica* are seen to lower the occurrence of cardiovascular diseases such as cardiac hypertrophy, myocardial ischaemia, atherosclerosis and hypertension.^125^ Asiaticoside is reported to protect cardiomyocytes against myocardial oxygen–glucose deprivation/reoxygenation (OGD/R) injury.^126^ Madecassoside has a protective effect on isolated rat hearts and isolated cardiomyocytes against reperfusion injury in vitro, as well as in vivo; it is seen to protect rat models against myocardial ischaemia–reperfusion injury‐induced infarction through its anti‐inflammatory, anti‐oxidative and anti‐apoptosis properties.^127^

Acute pancreatitis is characterized by tissue necrosis. Asiaticoside is proved to protect against mild acute pancreatitis (MAP) in caerulein‐induced MAP models by inhibiting pancreatic acinar cells necrosis, ameliorating pancreatitis‐associated histopathological changes, and lowering the severity of pancreatic tissue injury as well as pancreatitis‐associated lung injury in a severe acute pancreatitis model.^128^ Madecassoside improves insulin sensitivity in pancreatic cells to promote glucose‐stimulated insulin secretion (GSIS) and to enhance expression of insulin signalling proteins without any cytotoxic effects, making it a favourable drug compound in targeting type 2 diabetes mellitus.^129^

### 5.1 Anti‐inflammatory, anti‐allergic and other immunomodulatory properties

A majority of the medicinal activity of asiaticoside and madecassoside can be attributed to their regulation of inflammatory immune responses. As mentioned previously, *C. asiatica* shows significant immunosuppressive action in the context of atopic dermatitis, where it suppresses the infiltration of immune cells and controls inflammation.^82^ Asiaticoside exhibits anti‐inflammatory activity in regulating numerous diseases. According to Fong et al., asiaticoside might be a potential therapeutic agent for oedema prevention in inflammatory diseases like atherosclerosis because it can prevent endothelial barrier dysfunction elicited by the pro‐atherogenic cytokine, TNF‐α.^130^ Asiaticoside also shows protective effects against sepsis‐induced acute kidney injury by inhibiting inflammation.^131^

One of the best pieces of evidence for the anti‐inflammatory activity of these phytocompounds, particularly madecassoside, is protection against arthritis. Madecassoside shows further anti‐inflammation effects in monosodium urate (MSU)‐stimulated gouty arthritis and peritonitis. Gout is an inflammatory joint disease induced by MSU crystals accumulation, which is accompanied by the infiltration of neutrophils into joint spaces. Continued accumulation can lead to irreversible damage of the joint tissues and increases the risk of chronic inflammation.^132^ In a study by Lu et al., madecassoside suppressed MSU‐induced inflammation in mice with gouty arthritis, alleviated neutrophil infiltration and inhibited the secretion of pro‐inflammatory mediators in mice with peritoneal inflammation.^133^ Similar anti‐inflammatory benefits of madecassoside have been observed in mice with collagen‐induced arthritis; Li et al. demonstrated that the madecassoside‐treated arthritic mice showed improvement in the pathological damage to joint tissues, significant suppression of the pro‐inflammatory molecules COX‐2, PGE (2), TNF‐alpha and IL‐6, as well as upregulation of the anti‐inflammatory mediator IL‐10.^134^ Madecassoside is also seen to prevent the invasion of rheumatoid fibroblast‐like synoviocytes in rheumatoid arthritis by suppressing the NF‐κB pathway‐mediated inflammation.^135^ Furthermore, multiple studies verify the role of orally administered madecassoside in protecting against arthritis by similar action of inhibiting pro‐inflammatory cytokines and upregulating anti‐inflammatory ones.^136^, ^137^, ^138^ In a recent study, asiaticoside has been tested as an anti‐inflammatory and immunomodulatory agent during in vivo implantation of polylactic‐co‐glycolic acid fibrous scaffolds. Electro‐spun PLGA fibrous scaffolds are currently being tested in tissue engineering for bone, cartilage, skin and neural regeneration, as well as for drug delivery systems; however, the accumulation of degradation products due to implantation causes host inflammatory response triggered by the innate immune cells (dendritic cells, mast cells, granulocytes and macrophages) which hinders tissue regeneration. Based on varying microenvironments, macrophages can be polarized to form different phenotypes (M1 or M2) with different functions; the reversibility of polarization has critical therapeutic value, especially in diseases in which M1/M2 imbalance plays a pathogenic role. Asiaticoside can successfully suppress the expression of M1 (inflammatory) macrophages and inhibit the production of pro‐inflammatory cytokines, while simultaneously promoting the expression of M2 macrophages, which consistently release anti‐inflammatory cytokines. This is crucial in blocking host inflammatory response and achieving desirable implantation results, and asiaticoside is a favourable option for an anti‐inflammatory drug.^139^, ^140^

Asiaticoside also shows antiallergic properties. Mast cells which are associated with allergic inflammation, are immune cells that express a wide variety of membrane receptors involved in both the innate and the acquired immune responses. The main receptors are FcεRI, Toll‐like receptors, complement receptors and IgG receptors, which are involved in the activation of mast cells. Mast cells play a major role in the response to infections and are the main effectors in mediating response to allergens by degranulation and the release of histamine, pro‐inflammatory cytokines, chemokines and other vasoactive mediators.^141^ Asiaticoside can suppress allergic inflammation by blocking histamine release and mitigating mast cell degranulation, as well as reducing the generation of antigen‐induced inflammatory factors, via the FcεRI‐dependent signalling pathways.^142^

Furthermore, asiaticoside acts as an anti‐inflammatory and antipyretic agent that has been studied to dose‐dependently suppress lipopolysaccharide (LPS)‐induced fever and inflammation in rats, presumably by the inhibition of pro‐inflammatory mediators (serum tumour necrosis factor (TNF)‐α, interleukin (IL)‐6, liver myeloperoxidase (MPO), brain cyclooxygenase‐2 (COX‐2)) and the upregulation of liver heme oxygenase‐1 (HO‐1) protein activity (critical in preventing vascular inflammation.)^143^, ^144^ Taking the abundant evidence of anti‐inflammatory properties of asiaticoside as well as its regulation of macrophage polarization, mast cell degranulation and fever‐associated cytokines, more future studies dedicated to the effects of this natural extract on other immune responses may be beneficial from a clinical perspective.

### 5.2 Antipathogenic activities

*Centella asiatica* extracts can be considered a favourable option for natural anti‐virulence drugs that can target the virulence of pathogens while maintaining their cell viability, which can help prevent the development of pathogenic resistance to antibiotics. The Centella triterpenoids can be considered as phytoanticipins against a wide range of bacterial, fungal and parasitic agents due to their antimicrobial activities, selective cytotoxicity and protective function against pathogenic infections.^3^, ^145^, ^146^

An in vitro study by Vasanth et al. illustrates the effect of asiaticoside in reducing the production of cholera toxins in different strains of *Vibrio cholerae*, showing promising vibriocidal activity that can be incorporated into revised treatments for cholera.^10^ Oral administration of asiaticoside has proved effective in treating the parasitic infection of visceral leishmaniasis caused by *Leishmania donovani* in infected mice models. Asiaticoside cleared almost all parasitic burdens in the liver and spleen as well as mediated a switch in the host from a Th2‐ to a Th1‐type immune response, accompanied by induction of TNF‐α‐mediated nitric oxide production, which are important factors involved in macrophage function in antileishmanial defence mechanisms.^38^ Little evidence exists for anti‐viral effects of asiaticoside or madecassoside extracted from *C. asiatica*; however, asiaticoside derived from *Hydrocotyle sibthorpioides* (Apiaceae) is observed to significantly reduce the viral DNA transcription and replication of Hepatitis B virus (HBV) without resulting in toxicity.^147^

## 6 CONCLUSION

A variety of *C. asiatica* extracts have been extensively used in traditional medicine due to the wide spectrum of pharmacological activities associated with these secondary metabolites. This review highlights the activities of asiaticoside and madecassoside, the major saponin constituents of *C. asiatica* extracts, and provides a comprehensive view of their range of properties that can be harnessed for improving human health. Due to the lack of any reported adverse effects of *C. asiatica* in any clinical studies so far, the plant is categorized as a Class 1 herb (one that can safely be consumed when used appropriately) in the Botanical Safety Handbook. As illustrated in this review, research from the past two decades indicates a significant potential role for asiaticoside and madecassoside to be incorporated into modern treatment strategies, specifically targeting neurological and dermatological diseases, as well as offer potent benefits to a host of other medicinal and cosmetic requirements. Further research and clinical studies conducted on these compounds can offer novel therapeutic options and enhance existing medical knowledge through naturally occurring sources.

## AUTHOR CONTRIBUTIONS

**Shinjini Bandopadhyay:** Conceptualization (supporting); investigation (equal); methodology (equal); writing – original draft (equal). **Sujata Mandal:** Formal analysis (supporting); writing – review and editing (supporting). **Mimosa Ghorai:** Data curation (supporting); writing – review and editing (supporting). **Niraj Kumar Jha:** Formal analysis (supporting); writing – review and editing (supporting). **Manoj Kumar:** Validation (supporting); writing – review and editing (supporting). **Radha:** Data curation (supporting); writing – review and editing (supporting). **Arabinda Ghosh:** Writing – review and editing (supporting). **Jarosław Proćków:** project administration (supporting); resources (supporting); supervision (supporting); validation (supporting); visualization (supporting); writing – review and editing (supporting). **José M. Pérez de la Lastra:** Funding acquisition (lead); methodology (equal); writing – review and editing (equal). **Abhijit Dey:** Conceptualization (lead); project administration (supporting); resources (supporting); supervision (lead); writing – review and editing (supporting).

## FUNDING INFORMATION

This research was funded by project APOGEO (Cooperation Program INTERREG‐MAC 2014‐2020, with European Funds for Regional Development‐FEDER, “Agencia Canaria de Investigación, Innovación y Sociedad de la Información (ACIISI) del Gobierno de Canarias”, project ProID2020010134, CajaCanarias, Project 2019SP43, and the State Plan for Scientific, Technical Research and Innovation 2021‐2023 from the Spanish Ministry of Science and Innovation (project PLEC2022‐009507).

## CONFLICT OF INTEREST

The authors declare no competing financial interests.

## Data Availability

Data sharing not applicable to this article as no datasets were generated or analysed during the current study.

## References

[1] Gray NE , Alcazar Magana A , Lak P , et al. *Centella asiatica* – phytochemistry and mechanisms of neuroprotection and cognitive enhancement. Phytochem Rev. 2018;17(1):161‐194. doi:10.1007/s11101-017-9528-y 31736679 PMC6857646

[2] Brinkhaus B , Lindner M , Schuppan D , Hahn EG . Chemical, pharmacological and clinical profile of the east Asian medical plant *Centella asiatica* . Phytomedicine. 2000;7(5):427‐448. doi:10.1016/s0944-7113(00)80065-3 11081995

[3] James JT , Dubery IA . Pentacyclic triterpenoids from the medicinal herb, *Centella asiatica* (L.) urban. Molecules. 2009;14(10):3922‐3941. doi:10.3390/molecules14103922 19924039 PMC6255425

[4] James J , Dubery I . Identification and quantification of triterpenoid Centelloids in *Centella asiatica* (L.) urban by densitometric TLC. J Planar Chromat. 2011;24:82‐87. doi:10.1556/JPC.24.2011.1.16

[5] Hashim P , Sidek H , Helan MH , Sabery A , Palanisamy UD , Ilham M . Triterpene composition and bioactivities of *Centella asiatica* . Molecules. 2011;16(2):1310‐1322. doi:10.3390/molecules16021310 21278681 PMC6259745

[6] Arora R , Kumar R , Agarwal A , Reeta KH , Gupta YK . Comparison of three different extracts of *Centella asiatica* for anti‐amnesic, antioxidant and anticholinergic activities: in vitro and in vivo study. Biomed Pharmacother. 2018;105:1344‐1352. doi:10.1016/j.biopha.2018.05.156 30021372

[7] Shukla SD , Bhatnagar M , Khurana S . Critical evaluation of ayurvedic plants for stimulating intrinsic antioxidant response. Front Neurosci. 2012;6:112. doi:10.3389/fnins.2012.00112 22855669 PMC3405414

[8] Sun B , Wu L , Wu Y , et al. Therapeutic potential of *Centella asiatica* and its triterpenes: a review. Front Pharmacol. 2020;11:568032. doi:10.3389/fphar.2020.568032 33013406 PMC7498642

[9] Dahanukar S , Kulkarni R , Rege N . Pharmacology of medicinal plants and natural products. Indian J Pharmacol. 2000;32:81‐118.

[10] Vasanth S , Mohanraj RS , Mandal J . In‐vitro study of the effect of *Centella asiatica* on cholera toxin production and the gene expression level of ctxA gene in vibrio cholerae isolates. J Ethnopharmacol. 2021;279:113930. doi:10.1016/j.jep.2021.113930 33596471

[11] Gohil KJ , Patel JA , Gajjar AK . Pharmacological review on *Centella asiatica*: a potential herbal cure‐all. Indian J Pharm Sci. 2010;72(5):546‐556. doi:10.4103/0250-474X.78519 21694984 PMC3116297

[12] Hossain S , Urbi Z , Karuniawati H , et al. *Andrographis paniculata* (Burm. f.) wall. Ex Nees: an updated review of phytochemistry, antimicrobial pharmacology, and clinical safety and efficacy. Life (Basel). 2021;11(4):348. 33923529 10.3390/life11040348 PMC8072717

[13] Fahad FI , Barua N , Islam MS , et al. Investigation of the pharmacological properties of *Lepidagathis hyalina* Nees through experimental approaches. Life (Basel). 2021;11(3):180. 33668978 10.3390/life11030180 PMC7996513

[14] Bari MS , Khandokar L , Haque E , et al. Ethnomedicinal uses, phytochemistry, and biological activities of plants of the genus Gynura. J Ethnopharmacol. 2021;271:113834. 33465439 10.1016/j.jep.2021.113834

[15] Moni JNR , Adnan M , Tareq AM , et al. Therapeutic potentials of *Syzygium fruticosum* fruit (seed) reflected into an Array of pharmacological assays and prospective receptors‐mediated pathways. Life (Basel). 2021;11(2):155. 33671381 10.3390/life11020155 PMC7921944

[16] Bristy TA , Barua N , Montakim Tareq A , et al. Deciphering the pharmacological properties of methanol extract of *Psychotria calocarpa* leaves by in vivo, in vitro and in silico approaches. Pharmaceuticals (Basel). 2020;13(8):183. 32781707 10.3390/ph13080183 PMC7463710

[17] Sinan KI , Akpulat U , Aldahish AA , et al. LC‐MS/HRMS analysis, anti‐cancer, anti‐enzymatic and anti‐oxidant effects of *Boerhavia diffusa* extracts: a potential raw material for functional applications. Antioxidants (Basel). 2021;10(12):2003. 34943106 10.3390/antiox10122003 PMC8698501

[18] Shovo MARB , Tona MR , Mouah J , et al. Computational and pharmacological studies on the antioxidant, thrombolytic, anti‐inflammatory, and analgesic activity of *Molineria capitulata* . Curr Issues Mol Biol. 2021;43(2):434‐456. 34206443 10.3390/cimb43020035 PMC8929091

[19] Freitas MA , Vasconcelos A , Gonçalves ECD , et al. Involvement of opioid system and TRPM8/TRPA1 channels in the Antinociceptive effect of *Spirulina platensis* . Biomolecules. 2021;11(4):592. 33920609 10.3390/biom11040592 PMC8074039

[20] Uddin Chy MN , Adnan M , Chowdhury MR , et al. Central and peripheral pain intervention by Ophiorrhizarugosa leaves: potential underlying mechanisms and insight into the role of pain modulators. J Ethnopharmacol. 2021;276:114182. 33964360 10.1016/j.jep.2021.114182

[21] Ahmed S , Khan H , Aschner M , Mirzae H , Küpeli Akkol E , Capasso R . Anticancer potential of furanocoumarins: mechanistic and therapeutic aspects. Int J Mol Sci. 2020;21(16):5622. 32781533 10.3390/ijms21165622 PMC7460698

[22] Küpeli Akkol E , Genç Y , Karpuz B , Sobarzo‐Sánchez E , Capasso R . Coumarins and Coumarin‐related compounds in pharmacotherapy of cancer. Cancers (Basel). 2020;12(7):1959. 32707666 10.3390/cancers12071959 PMC7409047

[23] Fernández J , Silván B , Entrialgo‐Cadierno R , et al. Antiproliferative and palliative activity of flavonoids in colorectal cancer. Biomed Pharmacother. 2021;143:112241. 34649363 10.1016/j.biopha.2021.112241

[24] Ağagündüz D , Şahin TÖ , Yılmaz B , Ekenci KD , Şehriban DÖ , Capasso R . Cruciferous vegetables and their bioactive metabolites: from prevention to novel therapies of colorectal cancer. Evid Based Complement Alternat Med. 2022;2022:1534083. 35449807 10.1155/2022/1534083 PMC9017484

[25] Goni O , Khan MF , Rahman MM , et al. Pharmacological insights on the antidepressant, anxiolytic and aphrodisiac potentials of Aglaonema hookerianum Schott. J Ethnopharmacol. 2021;268:113664. 33278545 10.1016/j.jep.2020.113664

[26] Vieira G , Cavalli J , Gonçalves ECD , et al. Antidepressant‐like effect of Terpineol in an inflammatory model of depression: involvement of the cannabinoid system and D2 dopamine receptor. Biomolecules. 2020;10(5):792. 32443870 10.3390/biom10050792 PMC7280984

[27] Akkol EK , Tatlı II , Karatoprak GŞ , et al. Is Emodin with anticancer effects completely innocent? Two sides of the coin. Cancers (Basel). 2021;13(11):2733. 34073059 10.3390/cancers13112733 PMC8198870

[28] Tabassum R , Vaibhav K , Shrivastava P , et al. *Centella asiatica* attenuates the neurobehavioral, neurochemical and histological changes in transient focal middle cerebral artery occlusion rats. Neurol Sci. 2013;34(6):925‐933. doi:10.1007/s10072-012-1163-1 22864972

[29] Chen SW , Wang WJ , Li WJ , et al. Anxiolytic‐like effect of asiaticoside in mice. Pharmacol Biochem Behav. 2006;85(2):339‐344. doi:10.1016/j.pbb.2006.08.017 17059844

[30] Wang L , Guo T , Guo Y , Xu Y . Asiaticoside produces an antidepressant‐like effect in a chronic unpredictable mild stress model of depression in mice, involving reversion of inflammation and the PKA/pCREB/BDNF signaling pathway. Mol Med Rep. 2020;22(3):2364‐2372. doi:10.3892/mmr.2020.11305 32705202 PMC7411460

[31] Li SQ , Xie YS , Meng QW , Zhang J , Zhang T . Neuroprotective properties of Madecassoside from *Centella asiatica* after hypoxic‐ischemic injury. Pak J Pharm Sci. 2016;29(6):2047‐2051. 28375122

[32] Sun T , Liu B , Li P . Nerve protective effect of Asiaticoside against ischemia‐hypoxia in cultured rat cortex neurons. Med Sci Monit. 2015;21:3036‐3041. doi:10.12659/MSM.894024 26447863 PMC4603616

[33] Hossain S , Hashimoto M , Katakura M , Al Mamun A , Shido O . Medicinal value of asiaticoside for Alzheimer's disease as assessed using single‐molecule‐detection fluorescence correlation spectroscopy, laser‐scanning microscopy, transmission electron microscopy, and in silico docking. BMC Complement Altern Med. 2015;15:118. doi:10.1186/s12906-015-0620-9 25880304 PMC4422550

[34] Xu CL , Qu R , Zhang J , Li LF , Ma SP . Neuroprotective effects of madecassoside in early stage of Parkinson's disease induced by MPTP in rats. Fitoterapia. 2013;90:112‐118. doi:10.1016/j.fitote.2013.07.009 23876367

[35] Wu F , Bian D , Xia Y , et al. Identification of major active ingredients responsible for burn wound healing of *Centella asiatica* herbs. Evid Based Complement Alternat Med. 2012;2012:848093. doi:10.1155/2012/848093 23346217 PMC3546525

[36] Shen X , Guo M , Yu H , Liu D , Lu Z , Lu Y . Propionibacterium acnes related anti‐inflammation and skin hydration activities of madecassoside, a pentacyclic triterpene saponin from *Centella asiatica* . Biosci Biotechnol Biochem. 2019;83(3):561‐568. doi:10.1080/09168451.2018.1547627 30452312

[37] Byakodi M , Bagewadi Z , Muddapur U . Phytoconstituents profiling and evaluation of antimicrobial and antioxidant attributes of methanolic extract of *Centella asiatica* . Res J Pharmaceut Biol Chem Sci. 2018;9(3):493‐500.

[38] Bhaumik SK , Paul J , Naskar K , Karmakar S , De T . Asiaticoside induces tumour‐necrosis‐factor‐α‐mediated nitric oxide production to cure experimental visceral leishmaniasis caused by antimony‐susceptible and ‐resistant Leishmania donovani strains. J Antimicrob Chemother. 2012;67(4):910‐920. doi:10.1093/jac/dkr575 22258930

[39] Han AR , Lee S , Han S , et al. Triterpenoids from the leaves of *Centella asiatica* inhibit ionizing radiation‐induced migration and invasion of human lung cancer cells. Evid Based Complement Alternat Med. 2020;2020:3683460. doi:10.1155/2020/3683460 33029164 PMC7532382

[40] GBD 2016 Neurology Collaborators . Global, regional, and national burden of neurological disorders, 1990‐2016: a systematic analysis for the global burden of disease study 2016. Lancet Neurol. 2019;18(5):459‐480. doi:10.1016/S1474-4422(18)30499-X 30879893 PMC6459001

[41] Sweeney MD , Sagare AP , Zlokovic BV . Blood‐brain barrier breakdown in Alzheimer disease and other neurodegenerative disorders. Nat Rev Neurol. 2018;14(3):133‐150. doi:10.1038/nrneurol.2017.188 29377008 PMC5829048

[42] Hanapi NA , Mohamad Arshad AS , Abdullah JM , Tengku Muhammad TS , Yusof SR . Blood‐brain barrier permeability of Asiaticoside, Madecassoside and Asiatic acid in porcine brain endothelial cell model. J Pharm Sci. 2021;110(2):698‐706. doi:10.1016/j.xphs.2020.09.015 32949562

[43] Weiss N , Miller F , Cazaubon S , Couraud PO . The blood‐brain barrier in brain homeostasis and neurological diseases. Biochim Biophys Acta. 2009;1788(4):842‐857. doi:10.1016/j.bbamem.2008.10.022 19061857

[44] Kadry H , Noorani B , Cucullo L . A blood‐brain barrier overview on structure, function, impairment, and biomarkers of integrity. Fluid Barrier CNS. 2020;17(1):69. doi:10.1186/s12987-020-00230-3 PMC7672931 33208141

[45] Desai BS , Monahan AJ , Carvey PM , Hendey B . Blood‐brain barrier pathology in Alzheimer's and Parkinson's disease: implications for drug therapy. Cell Transplant. 2007;16(3):285‐299. doi:10.3727/000000007783464731 17503739

[46] Banks WA . From blood‐brain barrier to blood‐brain interface: new opportunities for CNS drug delivery. Nat Rev Drug Discov. 2016;15(4):275‐292. doi:10.1038/nrd.2015.21 26794270

[47] Miao R , Xia LY , Chen HH , Huang HH , Liang Y . Improved classification of blood‐brain‐barrier drugs using deep learning. Sci Rep. 2019;9(1):8802. doi:10.1038/s41598-019-44773-4 31217424 PMC6584536

[48] Pardridge WM . The blood‐brain barrier: bottleneck in brain drug development. NeuroRx. 2005;2(1):3‐14. doi:10.1602/neurorx.2.1.3 15717053 PMC539316

[49] Daneman R , Prat A . The blood‐brain barrier. Cold Spring Harb Perspect Biol. 2015;7(1):a020412. doi:10.1101/cshperspect.a020412 25561720 PMC4292164

[50] Obermeier B , Daneman R , Ransohoff RM . Development, maintenance and disruption of the blood‐brain barrier. Nat Med. 2013;19(12):1584‐1596. doi:10.1038/nm.3407 24309662 PMC4080800

[51] Tiwari S , Atluri V , Kaushik A , Yndart A , Nair M . Alzheimer's disease: pathogenesis, diagnostics, and therapeutics. Int J Nanomedicine. 2019;14:5541‐5554. doi:10.2147/IJN.S200490 31410002 PMC6650620

[52] Mantzavinos V , Alexiou A . Biomarkers for Alzheimer's disease diagnosis. Curr Alzheimer Res. 2017;14(11):1149‐1154. doi:10.2174/1567205014666170203125942 28164766 PMC5684784

[53] Lotankar S , Prabhavalkar KS , Bhatt LK . Biomarkers for Parkinson's disease: recent advancement. Neurosci Bull. 2017;33(5):585‐597. doi:10.1007/s12264-017-0183-5 28936761 PMC5636742

[54] Hou Y , Dan X , Babbar M , et al. Ageing as a risk factor for neurodegenerative disease. Nat Rev Neurol. 2019;15(10):565‐581. doi:10.1038/s41582-019-0244-7 31501588

[55] Tarafdar A , Pula G . The role of NADPH oxidases and oxidative stress in neurodegenerative disorders. Int J Mol Sci. 2018;19(12):3824. doi:10.3390/ijms19123824 30513656 PMC6321244

[56] Kritsilis M , Rizou SV , Koutsoudaki PN , Evangelou K , Gorgoulis VG , Papadopoulos D . Ageing, cellular senescence and neurodegenerative disease. Int J Mol Sci. 2018;19(10):2937. doi:10.3390/ijms19102937 30261683 PMC6213570

[57] Trist BG , Hare DJ , Double KL . Oxidative stress in the aging substantia nigra and the etiology of Parkinson's disease. Aging Cell. 2019;18(6):e13031. doi:10.1111/acel.13031 31432604 PMC6826160

[58] Tönnies E , Trushina E . Oxidative stress, synaptic dysfunction, and Alzheimer's disease. J Alzheimer's Dis. 2017;57(4):1105‐1121. doi:10.3233/JAD-161088 28059794 PMC5409043

[59] Chen CL , Tsai WH , Chen CJ , Pan TM . *Centella asiatica* extract protects against amyloid β1‐40‐induced neurotoxicity in neuronal cells by activating the antioxidative defence system. J Tradit Complement Med. 2015;6(4):362‐369. doi:10.1016/j.jtcme.2015.07.002 27774420 PMC5067859

[60] Haleagrahara N , Ponnusamy K . Neuroprotective effect of *Centella asiatica* extract (CAE) on experimentally induced parkinsonism in aged Sprague‐Dawley rats. J Toxicol Sci. 2010;35(1):41‐47. doi:10.2131/jts.35.41 20118623

[61] Hafiz ZZ , Amin M , Johari James RM , Teh LK , Salleh MZ , Adenan MI . Inhibitory effects of raw‐extract *Centella asiatica* (RECA) on acetylcholinesterase, inflammations, and oxidative stress activities via in vitro and in vivo. Molecules. 2020;25(4):892. doi:10.3390/molecules25040892 32079355 PMC7070982

[62] Orhan IE , Atasu E , Senol FS , et al. Comparative studies on Turkish and Indian *Centella asiatica* (L.) urban (gotu kola) samples for their enzyme inhibitory and antioxidant effects and phytochemical characterization. Ind Crops Prod. 2013;47:316‐322. doi:10.1016/j.indcrop.2013.03.022

[63] Xu CL , Wang QZ , Sun LM , et al. Asiaticoside: attenuation of neurotoxicity induced by MPTP in a rat model of parkinsonism via maintaining redox balance and up‐regulating the ratio of Bcl‐2/Bax. Pharmacol Biochem Behav. 2012;100(3):413‐418. doi:10.1016/j.pbb.2011.09.014 22001429

[64] Sampath U , Janardhanam VA . Asiaticoside, a trisaccaride triterpene induces biochemical and molecular variations in brain of mice with parkinsonism. Transl Neurodegenerat. 2013;2(1):23. doi:10.1186/2047-9158-2-23 PMC4177538 24262283

[65] Stegner D , Klaus V , Nieswandt B . Platelets as modulators of cerebral ischemia/reperfusion injury. Front Immunol. 2019;10:2505. doi:10.3389/fimmu.2019.02505 31736950 PMC6838001

[66] Zhang C , Chen S , Zhang Z , et al. Asiaticoside alleviates cerebral ischemia‐reperfusion injury via NOD2/mitogen‐activated protein kinase (MAPK)/nuclear factor kappa B (NF‐κB) signaling pathway. Med Sci Monit. 2020;26:e920325. doi:10.12659/MSM.920325 32006420 PMC7009775

[67] Luo Y , Wang C , Li WH , et al. Madecassoside protects BV2 microglial cells from oxygen‐glucose deprivation/reperfusion‐induced injury via inhibition of the toll‐like receptor 4 signaling pathway. Brain Res. 2018;1679:144‐154. doi:10.1016/j.brainres.2017.11.030 29198964

[68] Zhou Y , Wang S , Zhao J , Fang P . Asiaticoside attenuates neonatal hypoxic‐ischemic brain damage through inhibiting TLR4/NF‐κB/STAT3 pathway. Ann Translation Med. 2020;8(10):641. doi:10.21037/atm-20-3323 PMC7290617 32566578

[69] Solstrand Dahlberg L , Becerra L , Borsook D , Linnman C . Brain changes after spinal cord injury, a quantitative meta‐analysis and review. Neurosci Biobehav Rev. 2018;90:272‐293. doi:10.1016/j.neubiorev.2018.04.018 29702136

[70] Fan L , Li X , Liu T . Asiaticoside inhibits neuronal apoptosis and promotes functional recovery after spinal cord injury in rats. J Mol Neurosci. 2020;70(12):1988‐1996. doi:10.1007/s12031-020-01601-z 32529536

[71] Luo Y , Fu C , Wang Z , Zhang Z , Wang H , Liu Y . Asiaticoside attenuates the effects of spinal cord injury through antioxidant and anti‐inflammatory effects, and inhibition of the p38‐MAPK mechanism. Mol Med Rep. 2015;12(6):8294‐8300. doi:10.3892/mmr.2015.4425 26458544

[72] Maron E , Nutt D . Biological markers of generalized anxiety disorder. Dialogues Clin Neurosci. 2017;19(2):147‐158. doi:10.31887/DCNS.2017.19.2/dnutt 28867939 PMC5573559

[73] Cuijpers P , Quero S , Dowrick C , Arroll B . Psychological treatment of depression in primary care: recent developments. Curr Psychiatry Rep. 2019;21(12):129. doi:10.1007/s11920-019-1117-x 31760505 PMC6875158

[74] Bandelow B , Michaelis S , Wedekind D . Treatment of anxiety disorders. Dialogues Clin Neurosci. 2017;19(2):93‐107. doi:10.31887/DCNS.2017.19.2/bbandelow 28867934 PMC5573566

[75] Jana U , Sur TK , Maity LN , Debnath PK , Bhattacharyya D . A clinical study on the management of generalized anxiety disorder with *Centella asiatica* . Nepal Med Coll J. 2010;12(1):8‐11. 20677602

[76] Wanasuntronwong A , Tantisira MH , Tantisira B , Watanabe H . Anxiolytic effects of standardized extract of *Centella asiatica* (ECa 233) after chronic immobilization stress in mice. J Ethnopharmacol. 2012;143(2):579‐585. doi:10.1016/j.jep.2012.07.010 22841896

[77] Le‐Niculescu H , Roseberry K , Gill SS , et al. Precision medicine for mood disorders: objective assessment, risk prediction, pharmacogenomics, and repurposed drugs. Mol Psychiatry. 2021;26(7):2776‐2804. doi:10.1038/s41380-021-01061-w 33828235 PMC8505261

[78] Miller AH , Raison CL . The role of inflammation in depression: from evolutionary imperative to modern treatment target. Nat Rev Immunol. 2016;16(1):22‐34. doi:10.1038/nri.2015.5 26711676 PMC5542678

[79] Jeon SW , Kim YK . The role of neuroinflammation and neurovascular dysfunction in major depressive disorder. J Inflamm Res. 2018;11:179‐192. doi:10.2147/JIR.S141033 29773951 PMC5947107

[80] Tundis R , Loizzo MR , Bonesi M , Menichini F . Potential role of natural compounds against skin aging. Curr Med Chem. 2015;22(12):1515‐1538. doi:10.2174/0929867322666150227151809 25723509

[81] Peng W , Novak N . Pathogenesis of atopic dermatitis. Clin Exp Allergy. 2015;45(3):566‐574. doi:10.1111/cea.12495 25610977

[82] Lee Y , Choi HK , N'deh K , et al. Inhibitory effect of *Centella asiatica* extract on DNCB‐induced atopic dermatitis in HaCaT cells and BALB/c mice. Nutrients. 2020;12(2):411. doi:10.3390/nu12020411 32033291 PMC7071208

[83] Wang Y , Li S , Li C . Perspectives of new advances in the pathogenesis of vitiligo: from oxidative stress to autoimmunity. Med Sci Monit. 2019;25:1017‐1023. doi:10.12659/MSM.914898 30723188 PMC6373225

[84] Ling Y , Gong Q , Xiong X , et al. Protective effect of madecassoside on H2O2‐induced oxidative stress and autophagy activation in human melanocytes. Oncotarget. 2017;8(31):51066‐51075. doi:10.18632/oncotarget.17654 28881630 PMC5584231

[85] Platsidaki E , Dessinioti C . Recent advances in understanding *Propionibacterium acnes* (*Cutibacterium acnes*) in acne. F1000Research. 2018;7:F1000 faculty Rev‐1953. doi:10.12688/f1000research.15659.1 PMC6305227 30613388

[86] Fisher GJ , Kang S , Varani J , et al. Mechanisms of photoaging and chronological skin aging. Arch Dermatol. 2002;138(11):1462‐1470. doi:10.1001/archderm.138.11.1462 12437452

[87] Haftek M , Mac‐Mary S , Le Bitoux MA , et al. Clinical, biometric and structural evaluation of the long‐term effects of a topical treatment with ascorbic acid and madecassoside in photoaged human skin. Exp Dermatol. 2008;17(11):946‐952. doi:10.1111/j.1600-0625.2008.00732.x 18503551

[88] Sawant O , Khan T . Management of periorbital hyperpigmentation: An overview of nature‐based agents and alternative approaches. Dermatol Ther. 2020;33(4):e13717. doi:10.1111/dth.13717 32472659

[89] Lee J , Jung E , Lee H , Seo Y , Koh J , Park D . Evaluation of the effects of a preparation containing asiaticoside on periocular wrinkles of human volunteers. Int J Cosmet Sci. 2008;30:167‐173. doi:10.1111/j.1468-2494.2008.00440.x 18452433

[90] Jung E , Lee JA , Shin S , Roh KB , Kim JH , Park D . Madecassoside inhibits melanin synthesis by blocking ultraviolet‐induced inflammation. Molecules. 2013;18(12):15724‐15736. doi:10.3390/molecules181215724 24352025 PMC6290557

[91] Bylka W , Znajdek‐Awiżeń P , Studzińska‐Sroka E , Brzezińska M . *Centella asiatica* in cosmetology. Postepy Dermatol Alergol. 2013;30(1):46‐49. doi:10.5114/pdia.2013.33378 24278045 PMC3834700

[92] Kongkaew C , Meesomperm P , Scholfield CN , Chaiwiang N , Waranuch N . Efficacy and safety of *Centella Asiatica* (L.) Urb. On wrinkles: a systematic review of published Data and network meta‐analysis. J Cosmet Sci. 2020;71(6):439‐454. 33413787

[93] Mari W , Alsabri SG , Tabal N , Younes S , Sherif A , Simman R . Novel insights on understanding of keloid scar: article review. J Am Coll Clin Wound Special. 2016;7(1–3):1‐7. doi:10.1016/j.jccw.2016.10.001 PMC5197049 28053861

[94] Singkhorn S , Tantisira MH , Tanasawet S , Hutamekalin P , Wongtawatchai T , Sukketsiri W . Induction of keratinocyte migration by ECa 233 is mediated through FAK/Akt, ERK, and p38 MAPK signaling. Phytother Res. 2018;32(7):1397‐1403. doi:10.1002/ptr.6075 29532532

[95] Unahabhokha T , Sucontphunt A , Nimmannit U , Chanvorachote P , Yongsanguanchai N , Pongrakhananon V . Molecular signalings in keloid disease and current therapeutic approaches from natural based compounds. Pharm Biol. 2015;53(3):457‐463. doi:10.3109/13880209.2014.918157 25331681

[96] Song J , Xu H , Lu Q , et al. Madecassoside suppresses migration of fibroblasts from keloids: involvement of p38 kinase and PI3K signaling pathways. Burns. 2012;38(5):677‐684. doi:10.1016/j.burns.2011.12.017 22360962

[97] Wu X , Bian D , Dou Y , et al. Asiaticoside hinders the invasive growth of keloid fibroblasts through inhibition of the GDF‐9/MAPK/Smad pathway. J Biochem Mol Toxicol. 2017;31(8):e21922. doi:10.1002/jbt.21922 28346732

[98] Lee J , Jung E , Kim Y , et al. Asiaticoside induces human collagen I synthesis through TGFbeta receptor I kinase (TbetaRI kinase)‐independent Smad signaling. Planta Med. 2006;72(4):324‐328. doi:10.1055/s-2005-916227 16557473

[99] Bahramsoltani R , Farzaei MH , Rahimi R . Medicinal plants and their natural components as future drugs for the treatment of burn wounds: an integrative review. Arch Dermatol Res. 2014;306(7):601‐617. doi:10.1007/s00403-014-1474-6 24895176

[100] Liu M , Dai Y , Li Y , et al. Madecassoside isolated from *Centella asiatica* herbs facilitates burn wound healing in mice. Planta Med. 2008;74(8):809‐815. doi:10.1055/s-2008-1074533 18484522

[101] Kimura Y , Sumiyoshi M , Samukawa K , Satake N , Sakanaka M . Facilitating action of asiaticoside at low doses on burn wound repair and its mechanism. Eur J Pharmacol. 2008;584(2–3):415‐423. doi:10.1016/j.ejphar.2008.02.036 18353310

[102] Somboonwong J , Kankaisre M , Tantisira B , Tantisira MH . Wound healing activities of different extracts of *Centella asiatica* in incision and burn wound models: an experimental animal study. BMC Complement Altern Med. 2012;12:103. doi:10.1186/1472-6882-12-103 22817824 PMC3492213

[103] Intararuchikul T , Teerapattarakan N , Rodsiri R , et al. Effects of *Centella asiatica* extract on antioxidant status and liver metabolome of rotenone‐treated rats using GC‐MS. Biomed Chromatogr. 2019;33(2):e4395. doi:10.1002/bmc.4395 30242859

[104] Duggina P , Kalla CM , Varikasuvu SR , Bukke S , Tartte V . Protective effect of centella triterpene saponins against cyclophosphamide‐induced immune and hepatic system dysfunction in rats: its possible mechanisms of action. J Physiol Biochem. 2015;71(3):435‐454. doi:10.1007/s13105-015-0423-y 26168711

[105] Zhang L , Li HZ , Gong X , et al. Protective effects of Asiaticoside on acute liver injury induced by lipopolysaccharide/D‐galactosamine in mice. Phytomedicine. 2010;17(10):811‐819. doi:10.1016/j.phymed.2010.01.008 20171071

[106] Wang W , Wu L , Li Q , et al. Madecassoside prevents acute liver failure in LPS/D‐GalN‐induced mice by inhibiting p38/NF‐κB and activating Nrf2/HO‐1 signaling. Biomed Pharmacother. 2018;103:1137‐1145. doi:10.1016/j.biopha.2018.04.162 29715757

[107] Park DW , Jeon H , Kwon JE , et al. Hepatoprotective effect of *Centella asiatica* 50% ethanol extract against acetaminophen‐induced acute liver injury in BALB/c mice. Toxicol Res. 2020;37(2):261‐275. doi:10.1007/s43188-020-00063-0 33868982 PMC8007681

[108] Suresh K , Shimoda LA . Lung circulation. Comprehens Phys Ther. 2016;6(2):897‐943. doi:10.1002/cphy.c140049 PMC7432532 27065170

[109] Nathan SD , Barbera JA , Gaine SP , et al. Pulmonary hypertension in chronic lung disease and hypoxia. Eur Respir J. 2019;53(1):1801914. doi:10.1183/13993003.01914-2018 30545980 PMC6351338

[110] Wang XB , Wang W , Zhu XC , et al. The potential of asiaticoside for TGF‐β1/Smad signaling inhibition in prevention and progression of hypoxia‐induced pulmonary hypertension. Life Sci. 2015;137:56‐64. doi:10.1016/j.lfs.2015.07.016 26209140

[111] Wang X , Cai X , Wang W , et al. Effect of asiaticoside on endothelial cells in hypoxia‐induced pulmonary hypertension. Mol Med Rep. 2018;17(2):2893‐2900. doi:10.3892/mmr.2017.8254 29257311 PMC5783505

[112] Markopoulos GS , Roupakia E , Tokamani M , et al. Roles of NF‐kappa B signaling in the regulation of miRNAs impacting on inflammation in cancer. Biomedicine. 2018;6(2):40. doi:10.3390/biomedicines6020040 PMC6027290 29601548

[113] Qiu J , Yu L , Zhang X , et al. Asiaticoside attenuates lipopolysaccharide‐induced acute lung injury via down‐regulation of NF‐κB signaling pathway. Int Immunopharmacol. 2015;26(1):181‐187. doi:10.1016/j.intimp.2015.03.022 25835778

[114] Peng LY , Shi HT , Yuan M , et al. Madecassoside protects against LPS‐induced acute lung injury via inhibiting TLR4/NF‐κB activation and blood‐air barrier permeability. Front Pharmacol. 2020;11:807. doi:10.3389/fphar.2020.00807 32581788 PMC7289980

[115] Luo J , Zhang T , Zhu C , et al. Asiaticoside might attenuate bleomycin‐induced pulmonary fibrosis by activating cAMP and Rap1 signalling pathway assisted by A2AR. J Cell Mol Med. 2020;24(14):8248‐8261. doi:10.1111/jcmm.15505 32548952 PMC7348182

[116] Lu GX , Bian DF , Ji Y , et al. Madecassoside ameliorates bleomycin‐induced pulmonary fibrosis in mice by downregulating collagen deposition. Phytother Res. 2014;28(8):1224‐1231. doi:10.1002/ptr.5120 24458872

[117] Dang JW , Lei XP , Li QP , Dong WB . Asiaticoside attenuates hyperoxia‐induced lung injury in vitro and in vivo. Iran J Basic Med Sci. 2019;22(7):797‐805. doi:10.22038/ijbms.2019.35913.8556 32373302 PMC7196350

[118] Panizo S , Martínez‐Arias L , Alonso‐Montes C , et al. Fibrosis in chronic kidney disease: pathogenesis and consequences. Int J Mol Sci. 2021;22(1):408. doi:10.3390/ijms22010408 33401711 PMC7795409

[119] Zhang M , Liu S , Fang L , Wang G , Yin L . Asiaticoside inhibits renal fibrosis development by regulating the miR‐142‐5p/ACTN4 axis. Biotechnol Appl Biochem. 2021;69:313‐322. doi:10.1002/bab.2110 33444480

[120] Bose M , Almas S , Prabhakar S . Wnt signaling and podocyte dysfunction in diabetic nephropathy. J Investig Med. 2017;65(8):1093‐1101. doi:10.1136/jim-2017-000456 28935636

[121] Wang Z , Liu J , Sun W . Effects of asiaticoside on levels of podocyte cytoskeletal proteins and renal slit diaphragm proteins in adriamycin‐induced rat nephropathy. Life Sci. 2013;93(8):352‐358. doi:10.1016/j.lfs.2013.07.010 23871990

[122] Masola B , Oguntibeju OO , Oyenihi AB . *Centella asiatica* ameliorates diabetes‐induced stress in rat tissues via influences on antioxidants and inflammatory cytokines. Biomed Pharmacother. 2018;101:447‐457. doi:10.1016/j.biopha.2018.02.115 29501767

[123] Zhu Q , Zeng J , Li J , et al. Effects of compound Centella on oxidative stress and Keap1‐Nrf2‐ARE pathway expression in diabetic kidney disease rats. Evid Based Complement Alternat Med. 2020;2020:9817932. doi:10.1155/2020/9817932 32595756 PMC7277064

[124] Su Z , Ye J , Qin Z , Ding X . Protective effects of madecassoside against doxorubicin induced nephrotoxicity in vivo and in vitro. Sci Rep. 2015;5:18314. doi:10.1038/srep18314 26658818 PMC4677317

[125] Razali N , Ng CT , Fong LY . Cardiovascular protective effects of *Centella asiatica* and its triterpenes: a review. Planta Med. 2019;85(16):1203‐1215. doi:10.1055/a-1008-6138 31539918

[126] Zhang J , Yao M , Jia X , Xie J , Wang Y . Hexokinase II upregulation contributes to Asiaticoside‐induced protection of H9c2 Cardioblasts during oxygen‐glucose deprivation/Reoxygenation. J Cardiovasc Pharmacol. 2020;75(1):84‐90. doi:10.1097/FJC.0000000000000754 31569121

[127] Bian GX , Li GG , Yang Y , et al. Madecassoside reduces ischemia‐reperfusion injury on regional ischemia induced heart infarction in rat. Biol Pharm Bull. 2008;31(3):458‐463. doi:10.1248/bpb.31.458 18310910

[128] Wu K , Yao G , Shi X , et al. Asiaticoside ameliorates acinar cell necrosis in acute pancreatitis via toll‐like receptor 4 pathway. Mol Immunol. 2021;130:122‐132. doi:10.1016/j.molimm.2020.11.018 33308902

[129] Elhassan S , Candasamy M , Ching TS , Heng YK , Bhattamisra SK . Effect of madecassoside and catalpol in amelioration of insulin sensitivity in pancreatic (INS‐1 E) β‐cell line. Nat Prod Res. 2019;35:1‐5. doi:10.1080/14786419.2019.1696794 31797687

[130] Fong LY , Ng CT , Zakaria ZA , et al. Asiaticoside inhibits TNF‐α‐induced endothelial Hyperpermeability of human aortic endothelial cells. Phytother Res. 2015;29(10):1501‐1508. doi:10.1002/ptr.5404 26171791

[131] Zheng J , Zhang L , Wu M , Li X , Zhang L , Wan J . Protective effects of asiaticoside on sepsis‐induced acute kidney injury in mice. China J Chin Mater Med. 2010;35(11):1482‐1485. 20822026

[132] Khameneh HJ , Ho AW , Laudisi F , et al. C5a regulates IL‐1β production and leukocyte recruitment in a murine model of monosodium urate crystal‐induced peritonitis. Front Pharmacol. 2017;8:10. doi:10.3389/fphar.2017.00010 28167912 PMC5253373

[133] Lu X , Zeng R , Lin J , et al. Pharmacological basis for use of madecassoside in gouty arthritis: anti‐inflammatory, anti‐hyperuricemic, and NLRP3 inhibition. Immunopharmacol Immunotoxicol. 2019;41(2):277‐284. doi:10.1080/08923973.2019.1590721 31084401

[134] Li H , Gong X , Zhang L , et al. Madecassoside attenuates inflammatory response on collagen‐induced arthritis in DBA/1 mice. Phytomedicine. 2009;16(6–7):538‐546. doi:10.1016/j.phymed.2008.11.002 19135346

[135] Yu WG , Shen Y , Wu JZ , Gao YB , Zhang LX . Madecassoside impedes invasion of rheumatoid fibroblast‐like synoviocyte from adjuvant arthritis rats via inhibition of NF‐κB‐mediated matrix metalloproteinase‐13 expression. Chin J Nat Med. 2018;16(5):330‐338. doi:10.1016/S1875-5364(18)30064-5 29860993

[136] Dou Y , Luo J , Yu J , Xia Y , Dai Y . Cholinergic system is involved in the therapeutic effect of madecassoside on collagen‐induced arthritis in rats. Int Immunopharmacol. 2019;75:105813. doi:10.1016/j.intimp.2019.105813 31404889

[137] Wang T , Wei Z , Dou Y , et al. Intestinal interleukin‐10 mobilization as a contributor to the anti‐arthritis effect of orally administered madecassoside: a unique action mode of saponin compounds with poor bioavailability. Biochem Pharmacol. 2015;94(1):30‐38. doi:10.1016/j.bcp.2015.01.004 25600909

[138] Qiao S , Lian X , Yue M , et al. Regulation of gut microbiota substantially contributes to the induction of intestinal Treg cells and consequent anti‐arthritis effect of madecassoside. Int Immunopharmacol. 2020;89(Pt A):107047. doi:10.1016/j.intimp.2020.107047 33039960

[139] Funes SC , Rios M , Escobar‐Vera J , Kalergis AM . Implications of macrophage polarization in autoimmunity. Immunology. 2018;154(2):186‐195. doi:10.1111/imm.12910 29455468 PMC5980179

[140] Huang J , Zhou X , Shen Y , et al. Asiaticoside loading into polylactic‐co‐glycolic acid electrospun nanofibers attenuates host inflammatory response and promotes M2 macrophage polarization. J Biomed Mater Res A. 2020;108(1):69‐80. doi:10.1002/jbm.a.36793 31496042

[141] González‐de‐Olano D , Álvarez‐Twose I . Mast cells as key players in allergy and inflammation. J Investig Allergol Clin Immunol. 2018;28(6):365‐378. doi:10.18176/jiaci.0327 30530385

[142] Jiang JZ , Ye J , Jin GY , et al. Asiaticoside mitigates the allergic inflammation by abrogating the degranulation of mast cells. J Agric Food Chem. 2017;65(37):8128‐8135. doi:10.1021/acs.jafc.7b01590 28891650

[143] Wan J , Gong X , Jiang R , Zhang Z , Zhang L . Antipyretic and anti‐inflammatory effects of asiaticoside in lipopolysaccharide‐treated rat through up‐regulation of heme oxygenase‐1. Phytother Res. 2013;27(8):1136‐1142. doi:10.1002/ptr.4838 22972613

[144] Araujo JA , Zhang M , Yin F . Heme oxygenase‐1, oxidation, inflammation, and atherosclerosis. Front Pharmacol. 2012;3:119. doi:10.3389/fphar.2012.00119 22833723 PMC3400084

[145] Soyingbe OS , Mongalo NI , Makhafola TJ . In vitro antibacterial and cytotoxic activity of leaf extracts of *Centella asiatica* (L.) Urb, Warburgia salutaris (Bertol. F.) Chiov and Curtisia dentata (Burm. F.) C.A.Sm ‐ medicinal plants used in South Africa. BMC Complement Alternat Med. 2018;18(1):315. doi:10.1186/s12906-018-2378-3 PMC6267026 30497461

[146] Dash B , Faruquee H , Biswas S , Alam M , Sisir S , Prodhan UK . Antibacterial and antifungal activities of several extracts of *Centella asiatica* L. against some human pathogenic microbes. Life Sci Med Res. 2011:1‐5.

[147] Huang Q , Zhang S , Huang R , et al. Isolation and identification of an anti‐hepatitis B virus compound from Hydrocotyle sibthorpioides lam. J Ethnopharmacol. 2013;150(2):568‐575. doi:10.1016/j.jep.2013.09.009 24051027

[148] Monton C , Settharaksa S , Luprasong C , Songsak T . An optimization approach of dynamic maceration of *Centella asiatica* to obtain the highest content of four centelloids by response surface methodology. Rev Brasil Farmacogn. 2019;29(2):254‐261. doi:10.1016/j.bjp.2019.01.001

[149] Hiranvarachat B , Devahastin S , Soponronnarit S . Comparative evaluation of atmospheric and vacuum microwave‐assisted extraction of bioactive compounds from fresh and dried *Centella asiatica* L. leaves. Int J Food Sci Technol. 2015;50:750‐757. doi:10.1111/ijfs.12669

[150] Sen KK , Chouhan KBS , Tandey R , Mehta R , Mandal V . Impact of microwaves on the extraction yield of phenolics, flavonoids, and triterpenoids from Centella leaves: An approach toward digiti botanical extraction. Pharmacogn Mag. 2019;15:S267‐S273. doi:10.4103/pm.pm_99_19

[151] Rafamantanana MH , Rozet E , Raoelison GE , et al. An improved HPLC‐UV method for the simultaneous quantification of triterpenic glycosides and aglycones in leaves of *Centella asiatica* (L.) Urb (APIACEAE). J Chromatogr B. 2009;877(23):2396‐2402. doi:10.1016/j.jchromb.2009.03.018 19349219

[152] Shen Y , Liu A , Ye M , et al. Analysis of biologically active constituents in *Centella asiatica* by microwave‐assisted extraction combined with LC–MS. Chromatographia. 2009;70:431‐438. doi:10.1365/s10337-009-1152-6

[153] Randriamampionona D , Diallo B , Rakotoniriana F , et al. Comparative analysis of active constituents in *Centella asiatica* samples from Madagascar: application for ex situ conservation and clonal propagation. Fitoterapia. 2007;78(7–8):482‐489. doi:10.1016/j.fitote.2007.03.016 17560738

[154] Niamnuy C , Charoenchaitrakool M , Mayachiew P , Devahastin S . Bioactive compounds and bioactivities of *Centella asiatica* (L.) urban prepared by different drying methods and conditions. Dry Technol Int J. 2013;31:2007‐2015. doi:10.1080/07373937.2013.839563

[155] Kim W‐J , Kim J , Veriansyah B , et al. Extraction of bioactive components from *Centella asiatica* using subcritical water. J Supercrit Fluid. 2009;48(3):211‐216. doi:10.1016/j.supflu.2008.11.007

